# Solvent and Temperature Effects on Photoinduced Proton-Coupled Electron Transfer in the Marcus Inverted Region

**DOI:** 10.1021/acs.jpca.1c05764

**Published:** 2021-08-25

**Authors:** Laura F. Cotter, Belinda Pettersson Rimgard, Giovanny A. Parada, James M. Mayer, Leif Hammarström

**Affiliations:** † Department of Chemistry, Yale University, New Haven, Connecticut 06520, United States; ‡ Department of Chemistry − Ångström Laboratory, Uppsala University, Box 523, SE75120 Uppsala, Sweden

## Abstract

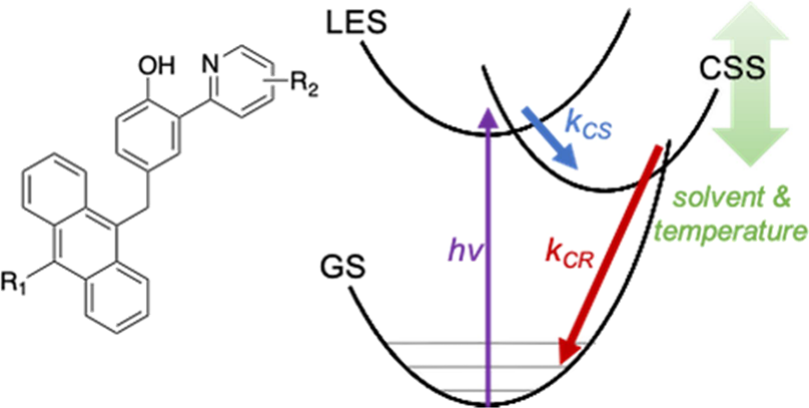

Concerted proton-coupled electron transfer (PCET) in the Marcus inverted region was recently demonstrated (*Science***2019**, *364*, 471–475). Understanding the requirements for such reactivity is fundamentally important and holds promise as a design principle for solar energy conversion systems. Herein, we investigate the solvent polarity and temperature dependence of photoinduced proton-coupled charge separation (CS) and charge recombination (CR) in anthracene–phenol–pyridine triads: **1** (10-(4-hydroxy-3-(4-methylpyridin-2-yl)benzyl)anthracene-9-carbonitrile) and **2** (10-(4-hydroxy-3-(4-methoxypyridin-2-yl)benzyl)anthracene-9-carbonitrile). Both the CS and CR rate constants increased with increasing polarity in acetonitrile:*n*-butyronitrile mixtures. The kinetics were semi-quantitatively analyzed where changes in dielectric and refractive index, and thus consequently changes in driving force (−Δ*G*°) and reorganization energy (λ), were accounted for. The results were further validated by fitting the temperature dependence, from 180 to 298 K, in *n*-butyronitrile. The analyses support previous computational work where transitions to proton vibrational excited states dominate the CR reaction with a distinct activation free energy (ΔG*_CR_ ∼ 140 meV). However, the solvent continuum model fails to accurately describe the changes in Δ*G*° and λ with temperature via changes in dielectric constant and refractive index. Satisfactory modeling was obtained using the results of a molecular solvent model [*J. Phys. Chem. B***1999**, *103*, 9130–9140], which predicts that λ decreases with temperature, opposite to that of the continuum model. To further assess the solvent polarity control in the inverted region, the reactions were studied in toluene. Nonpolar solvents decrease both Δ*G*°_CR_ and λ, slowing CR into the nanosecond time regime for **2** in toluene at 298 K. This demonstrates how PCET in the inverted region may be controlled to potentially use proton-coupled CS states for efficient solar fuel production and photoredox catalysis.

## Introduction

The thermochemistry and kinetics of electron transfer (ET) and proton transfer (PT) are often intimately correlated in processes known as proton-coupled electron transfer (PCET) reactions.^1−10^ These associated electron–proton transfer reactions are critical to numerous fundamental energy conversion processes, from photosynthesis and respiration to combustion and fuel cells. Such processes may become even more favorable when high-energy intermediates can be bypassed via a concerted mechanism, where PT and ET occur in a single kinetic step (CPET).

Marcus theory, in its most archetypical form, predicts ET rate constants based on the reaction free-energy barrier (ΔG*), which depends on the intrinsic reorganization energy (λ) and the reaction driving force (−Δ*G*°):^11^
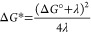
1

Subsequent efforts by Levich, Jortner, Marcus, and others led to the development of a quantum mechanical description of ET reactions and rates.^12−14^ In the high-temperature limit, all solvent and solute modes can be treated classically, and one obtains the following expression for the nonadiabatic rate constant:
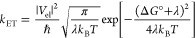
2where *V*_el_ is the electronic coupling between the reactants and products, *k*_B_ is the Boltzmann constant, and *T* is the temperature. As evident in eq 2, a remarkable prediction of Marcus theory is the bell-shaped free-energy dependence. Due to the quadratic relationship between *k*_ET_ and the driving force, the rate constant reaches a maximum when −Δ*G°* = λ and then, counterintuitively, proceeds to decrease with a further increase in driving force. This regime, where −Δ*G°* > λ, is known as the Marcus inverted region (MIR).

Development of theories for ET and PT has been combined and extended to describe PCET reactions, treating the proton quantum mechanically;^15−20^ for a review of this development, see ref (3). The following expression has been obtained for nonadiabatic CPET in the high-temperature limit:^21^
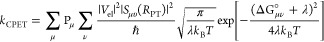
3where the summations are over transitions between different proton vibrational states of the reactants (μ) and products (ν) weighted by the Boltzmann population (*P*_μ_) of a given reactant proton vibrational state. The coupling can be approximated by the electronic coupling, *V*_el_, multiplied by the proton vibrational wavefunction overlap between reactant and product states (*S*_μν_). Thermal fluctuations result in a distribution of proton donor–acceptor distances, typically on the scale of 0.1–0.3 Å;^22−26^ thus, *S*_μν_ is an integral over various PT distances (*R*_PT_).

Decades after the formulation of the Marcus theory, the first widely accepted experimental evidence of ET reactions in the MIR was obtained in the mid-1980s by Closs and Miller and co-workers for ground-state (GS) charge shift reactions.^27,28^ This was closely followed by a report of inverted ET in photoinduced charge recombination (CR) reactions by Wasielewski and co-workers.^29^ Specific details of the rate versus free-energy correlations in the MIR indicated the involvement of nuclear tunneling of medium-frequency modes (typically aromatic C–C vibrations).^11^ Contributions from such medium-frequency modes attenuate the inverted region effect, leading to a shallower decrease of *k*_ET_ with increasing driving force than would be predicted from eq 2. A similar effect can be expected for CPET reactions due to contributions from high-frequency proton vibrationally excited states. The large electron–proton coupling, that is, the large shift in the equilibrium nuclear distance between reactants and products, was proposed to make nuclear tunneling to higher states even more important for CPET compared to the typical case of ET. Accordingly, it was originally predicted that the inverted region behavior would not be observed for CPET, even for extremely exergonic reactions.^22,30^

**Scheme 1:**
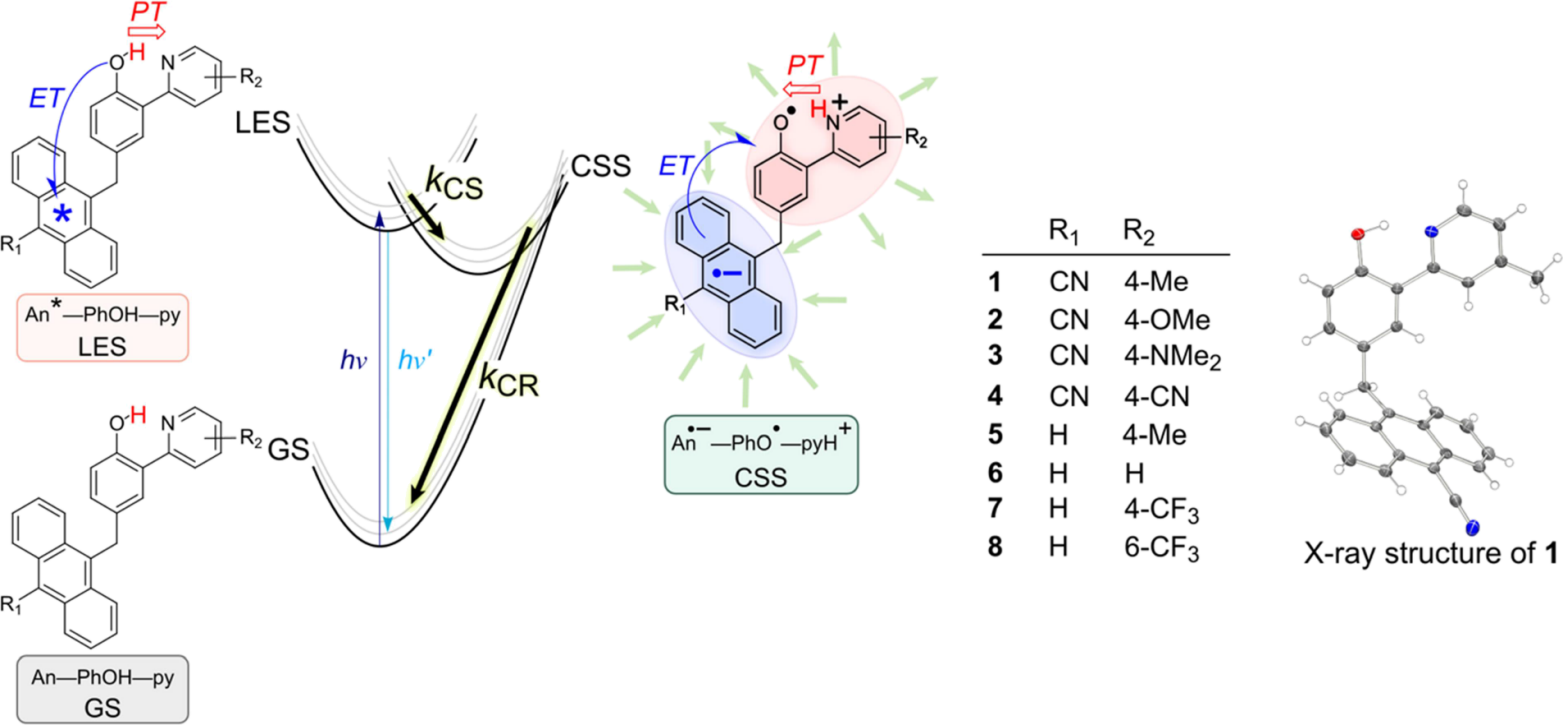
Structures of Anthracene–Phenol–Pyridine Triads **1–8** and Reaction Scheme for Photoinduced *e*^–^/H^+^ Charge Separation (CS) and CR Adapted from ref (31). Copyright the American Association for the Advancement of Science, 2019.

In contrast to these predictions, we recently reported CPET reactions exhibiting the inverted region behavior within a series of anthracene–phenol–pyridine (An-PhOH-py) molecular triads **1–8** (Scheme 1).^31^ The series was designed to vary Δ*G°* for photoinduced, proton-coupled CS and CR by substitution effects on the anthracene (electron acceptor) and pyridine (proton acceptor) moieties. Light excitation of the anthracene unit triggers ET from phenol to anthracene, concerted with PT of the phenolic proton to pyridine. The CS rate constant (*k*_CS_) increases with increasing driving force as expected for a reaction in the normal region (eqs 2 and 3, −Δ*G°* < λ). The subsequent CR reaction for **1**–**3** reforms the GS reactants in a CPET reaction in the MIR (−Δ*G°* > λ). The CR rate constants (*k*_CR_) decrease with increasing driving force within the series (**1** < **2** < **3**) as well as when the solvent polarity decreases from dimethylformamide (DMF, ε_s_ = 38.25) to dichloromethane (DCM, ε_s_ = 8.93).^32^ The strong solvent dependence is indicative of an inverted reaction since the effects of decreasing polarity on Δ*G°* and λ of a CR inverted reaction both result in shifting the reaction deeper into the MIR. Moreover, in spite of the earlier general predictions (see above), the observed inverted CR rates were satisfactorily modeled using the theory of Hammes-Schiffer and co-workers (eq 3), with contributions from proton vibrational excited states in the electronic GS products.^31,33^ The modeling used a description of the proton potentials as double wells that was more accurate in this case than the anharmonic or Morse potentials used for the previous general predictions.^30^

In the original Marcus model for ET, the inverted region stems from the increase in the activation barrier with increasing driving force once −Δ*G°* > λ. Quantum mechanical models^12−14^ provide an alternative view of the MIR, where activationless reactions to high product vibrational levels have a smaller vibrational wavefunction overlap when −Δ*G°* becomes larger. This is analogous to the origin of the energy gap law for nonradiative transitions. This would lead to the prediction of an inverted region ET rate constant that is independent, or nearly independent, of temperature.^34−36^ Theoretical modeling of the CR reactions in **1**–**3** in DCM according to eq 3 instead suggested that the dominant contributing reactant-to-product vibronic transitions (mainly around μ = 0 → ν = 3) indeed have a significant barrier (ΔG* ≈ 90 meV in DCM), with −Δ*G*°_μν_ larger than λ (see the Supporting Information in ref (31)). The barrierless transition (0 → 7) with −Δ*G*°_μν_ ≅ λ, on the other hand, has a negligible proton vibrational wavefunction overlap and therefore its contribution to *k*_CPET-CR_ is negligible. This explains why the MIR effect was observed for the CPET reactions of the An-PhOH-py triads.

Advancing the understanding of the conditions and parameters that allow for the MIR behavior in CPET reactions is of fundamental and general interest. Photoinduced CPET reactions in the MIR could enable the design of more efficient technologies for harvesting solar energy. This is particularly important for processes relying on the formation of charge-separated states (CSS) as energy-storing transient species, many of which implicate PCET steps, such as in photosynthesis. These highly reactive species are susceptible to energy-wasting CR reactions, which must be slow enough to allow for the productive, fuel-producing reactions to dominate. MIR kinetics, as proposed by Marcus^37^ and others, is thought to fulfill this function and is a fundamental principle operating in the primary CS reactions of photosynthesis. Although this hypothesis is widely accepted, it is rarely verified in natural systems.^38^ Therefore, it is important to understand what features allow for the MIR behavior and whether the MIR is a general phenomenon in CPET or if the An-PhOH-py triads are simply an exception to the rule.

The study presented here was designed to test and extend our understanding of inverted CPET in this system of An-PhOH-py triads. It focuses on the effects of temperature and solvent polarity on the photoinduced CPET reactivity of triads **1** and **2** using UV–vis femtosecond transient absorption (TA) spectroscopy. (Triad **3** also shows CR of the CSS with inverted region kinetics; however, it was excluded from the present studies due to its negligible solubility in nitrile solvents and toluene.) By using mixtures of similar solvents *n*-butyronitrile (PrCN) and acetonitrile (MeCN), the solvent polarity was systematically varied to study the effects of modulating Δ*G°* and λ of the reactions. Temperature-dependent experiments in PrCN probed the influences of Δ*G°*, λ, Marcus barriers, and thermal state populations. Experiments in toluene (Tol) were conducted to extend the investigation to a nonpolar solvent, which should maximize the inverted region effect for CR by minimizing the reorganization energy and maximizing the driving force.

These studies give further support for and insight into the MIR behavior of the An-PhOH-py triads. The results herein demonstrate that long-lived (1H^+^/1*e*^–^) CSSs can be achieved via slow MIR recombination kinetics and therefore such states could, in principle, participate in follow-up chemical reactions. This proof of principle could therefore be of assistance in developing solar to chemical energy conversion schemes.

## Experimental Details

Transient spectroscopy was performed with a 3 kHz 800 nm output of a Ti:sapphire amplifier (1.5 mJ, 45 fs FWHM, Libra, Coherent), which was split into pump (35%) and probe (65%). To achieve the 400 nm pump, 800 nm light was frequency-doubled using a 0.2 mm-thick BBO crystal (EKSMA Optics) prior to being chopped (1.5 kHz) in the sample compartment (Newport TAS). To avoid major effects of rotational depolarization, the pump was made pseudo-unpolarized using a depolarizer (Thorlabs). A white light supercontinuum probe was generated by focusing the light onto a 4 mm CaF_2_ crystal after passing through an 8 ns optical delay stage (Newport TAS). The probe spectra were recorded using a custom-made 200–1000 nm silicon diode array (Newport). Triads **1** and **2** from previous studies^31^ were dissolved in PrCN/MeCN (Sigma-Aldrich/Merck, ≥99.0% (GC)/spec. grade) mixtures, prepared in 1 mm × 10 mm quartz cuvettes, with an absorption of ∼0.1–0.15 at 400 nm measured using a Varian Cary 50 or 5000. The pump intensity was attenuated to 150 μW, and for each mixture, three scans were collected and averaged using 1000 ms integration time. Experiments with toluene (Tol, Merck, spectroscopic grade) followed the same procedure.

For temperature-dependent experiments in PrCN (Sigma-Aldrich, ≥99.0% (GC)), the solvent was dried overnight over molecular sieves (3 Å, 8–12 mesh, Sigma-Aldrich) and later filtered using Acrodisc 2 mm syringe filters (0.45 μm, WWPTFE membrane). The temperature was controlled using an Optistat DN1704 cryostat (Oxford Instruments NanoScience) with an ITC 501 controller. The cryostat was cooled using N_2(liq)_ and purged with N_2(gas)_. For each new temperature, 1 h of equilibration time was allowed. The samples were prepared in a long-necked 2 mm × 10 mm quartz cuvette with ∼0.4 absorption at 400 nm. The pump intensity was altered to 350 μW, and for each sample, three scans were averaged (1000 ms integration).

The collected spectra were fitted using Surface Xplorer (Ultrafast systems), the R package TIMP/Glotaran,^39,40^ as well as a home-made MATLAB script by Dr. J. Petersson^41^ and Dr. J. Föhlinger^100^ for global analysis of the nitrile mixtures and the temperature dependence in PrCN. Additionally, target analysis was used for the Tol data (K-matrix and compartment scheme are provided in the Supporting Information). Transient UV–vis spectra from ca. 410 to 760 nm were chirp-corrected for global analysis with a sequential model with three to four components. Due to the additional optical glass and sample pathlength in the cryostat, the initial artifact became more apparent in the time traces. To avoid its influence on the fitted parameters, the fitting was limited to times after 0.5–0.9 ps for the temperature-dependent data and after 0.5 ps for the nitrile mixtures.

## Results

### Overview of Triad Properties

Substituent effects on anthracene and pyridine of triads **1**–**8** vary the driving forces for CPET by ∼0.9 eV for CS and by ∼1.1 eV for CR in DCM.^31^ Specific structural features of the triads include a strong intramolecular hydrogen bond between phenol and pyridine as well as a methylene spacer to keep the anthracene and phenol–pyridine motifs electronically distinct. The X-ray structures of **1**, **3**, **5**, and **6** confirm that the anthracene and phenol–pyridine units lie out-of-plane relative to each other, and that the structure is sufficiently rigid to prevent intramolecular π–π stacking.^31,42^

**Figure 1:**
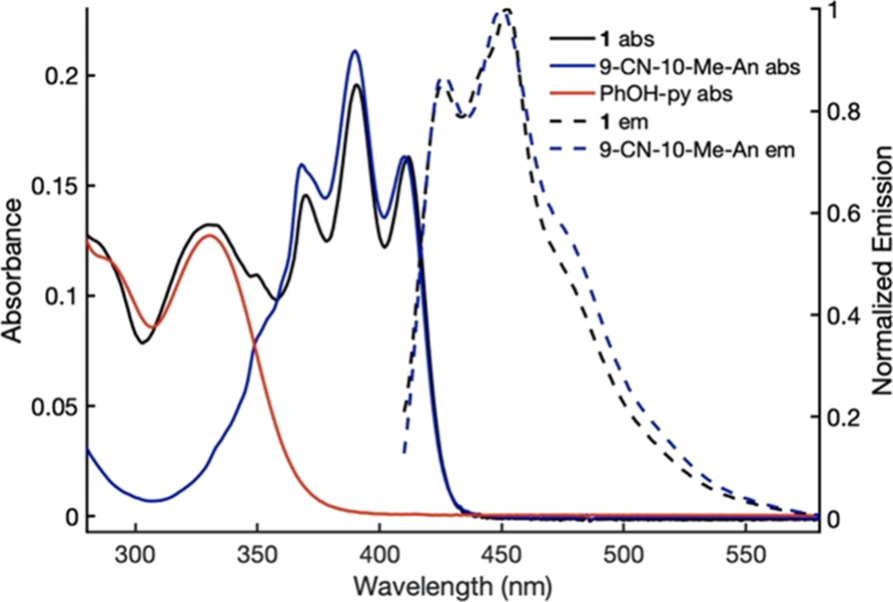
Absorption (solid lines) and fluorescence (dashed lines) spectra of **1** (0.020 ± 0.005 mM, black) and reference compounds 9-CN-10-Me-An (0.022 ± 0.05 mM, blue) and PhOH-py (0.015 ± 0.006 mM, red) in MeCN. Fluorescence spectra were recorded with 400 nm excitation. Figure redrawn from ref (31). Copyright the American Association for the Advancement of Science, 2019.

Figure 1 shows the steady-state absorption and fluorescence spectra of **1** measured in MeCN,^31^ which is representative of those of triad **2**. The reference spectra of the individual subunits in the same solvent are also included, specifically the absorption spectra of 2,4-di-tertbutyl-6-(pyridin-2-yl)phenol (PhOH-py) and the absorption and fluorescence spectra of 9-cyano-10-methylanthracene (9-CN-10-Me-An). The absorption spectrum of **1** indicates that the phenol–pyridine and anthracene subunits are weakly coupled as it agrees well with the sum of the spectra of the two subunits. The fluorescence spectra of **1** and those of 9-CN-10-Me-An are similar, but for the triad, the fluorescence yield is more than 1000 times smaller due to emission quenching by the phenol–pyridine unit.^42^ The absorption and emission of the 0 → 0 transition in anthracene overlap significantly in the spectra. Hence, the excited-state energy (*E*_0–0_, which approximates Δ*G*° of the locally excited state (LES) relative to the GS) can be estimated as 2.97 eV from the average wavenumber of the two 0 → 0 transition maxima. For **1** in PrCN, it was estimated that Δ*G°*_CS_ ∼ −0.49 eV and Δ*G°*_CR_ ∼ −2.48 eV, with the corresponding values for **2** being ∼ −0.54 eV and ∼ −2.43 eV, respectively.^31^

### TA in PrCN at Room Temperature

**Figure 2:**
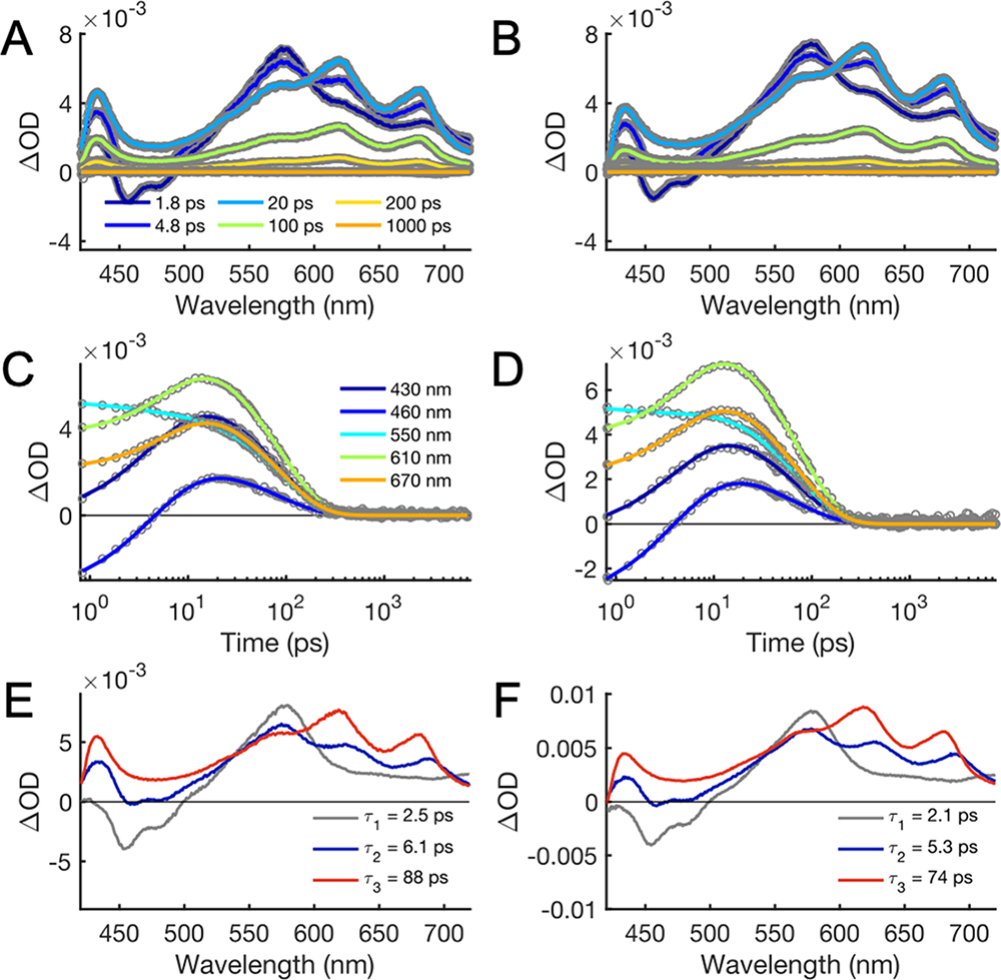
UV–vis TA data of **1** (A,C) and **2** (B,D) in PrCN at 298 K after fs excitation at 400 nm and results of global fits (E,F). (A,B): TA spectra at different times after excitation (gray) and fitted spectra (in color); (C,D): TA time traces at selected wavelengths (gray circles) and fitted traces (in color); and (E,F): evolution-associated spectra (EAS) from global analysis with a sum of three exponential components. The resulting time constants and assignment of the process are listed in Table 2. Residual plots from the fits are shown in the Supporting Information.

Figure 2A–D shows UV–vis TA data of **1** and **2** in PrCN at 298 K after excitation at 400 nm. This is representative of the observed spectral features for **1** and **2** in the PrCN/MeCN mixtures and in PrCN between 180 and 298 K (vide infra). Photoexcitation of the triads into the anthracene absorption band results in the initial formation of LES on the cyanoanthracene moiety (^1*^An-PhOH-py, Scheme 1). The LES is characterized by its stimulated emission (SE) from 410 to 500 nm and a broad excited-state absorption (ESA) above 500 nm with a peak at ca. 575 nm (dark blue TA spectrum in Figure 2A,B). ^1*^An is a strong oxidant that triggers (1H^+^/1*e^–^*) CS to form the CSS, An^•–^–PhO^•^–pyH^+^ on a time scale of ∼10 ps. The CSS consists of an anthracene radical anion, with positive bands at ca. 625 and 675 nm, a phenoxyl radical, with a band at ca. 425 nm, and pyridinium (light blue and green TA spectra in Figure 2A). These spectral features were previously characterized by a combination of steady-state and transient spectroscopies in the UV–vis and mid-IR range.^31^ The CSS features disappear on a time scale of ∼100 ps, leaving no significant TA signal after 1 ns.

A global fit of the TA data to a sum of exponential decays requires three time components to obtain satisfactory fits. For **1**, the time constants are τ_1_ = 2.5 ps, τ_2_ = 6.1 ps, and τ_3_ = 88 ps, while for **2**, the time constants are slightly smaller (Table 1). The resulting EAS for each component represent the absorption spectrum of the species corresponding to that time constant (note that the anthracene ground-state bleach is outside the spectral window shown in Figure 2). The EAS of the first two components each show SE (negative bands at 450–500 nm) and ESA (a positive band with maxima around 570 nm). Both spectral features are attributed to ^1*^An.^31^ The SE is red-shifted while the ESA is blue-shifted in the second component relative to the first one (Figure 2E,F). However, the second component additionally contains characteristic features of the CSS: broad absorption with peaks around 620 and 680 nm attributed to the An^•–^ radical and a narrower band around 425 nm attributed to the phenoxy radical.^31^ Thus, we conclude that the first component corresponds to the “hot” local ^1*^An state (LES_hot_) undergoing CPET to the CSS with τ_1_ = 2.5 ps in parallel with thermal relaxation. Thermal relaxation of “hot” excited states in solution is nonexponential,^43^ but given the competing CS reaction, the data do not justify a more complicated kinetic model. The relaxed LES then undergoes CS to form the CSS with τ_2_ = 6.1 ps. This explains the slight shift of SE and ESA between the first and second components and the biexponential generation of CSS. The EAS of the third component shows only the spectroscopic features of the CSS, which decays monotonically by CR to reform the GS with τ_3_ = 88 ps. The EAS An^•–^ features above 600 nm blueshift slightly from the second to third component indicating that the initial CPET from the LES_hot_ generates hot CSS (CSS_hot_) to some extent. In contrast, the narrow band around 425 nm, distinctive of PhO^•^, remains unshifted indicating that the excess thermal energy resides on the An^•–^ moiety. Relaxation of CSS_hot_ could not be resolved, but its expected TA changes are very small compared to those resulting from the CEPT from the relaxed LES. Therefore, we believe that the effect of the CSS_hot_ relaxation on τ_2_ is within the experimental and analytical errors. Triads **1** and **2** show qualitatively the same behavior (Figure 2). All time constants and their assignments are listed in Table 1.

**Table 1:** Time Constants^a^*^,^*^b^ and Assignments^c^ from Global Fits to the TA Data in PrCN between 180 and 298 K

	triad 1			triad 2		
time constants^a^*^,^*^b^	τ_1_ (ps)	τ_2_ (ps)	τ_3_ (ps)	τ_1_ (ps)	τ_2_ (ps)	τ_3_ (ps)
assignment^c^	VR + CS_hot_	CS	CR	VR + CS_hot_	CS	CR
180 K	5.6	38	142	4.7	30	134
200 K	4.9	25	106	4.4	21	102
220 K	3.8	16	96	3.3	14	83
240 K	3.8	11	88	2.7	9	78
260 K	2.9	8.7	86	3.2	8	77
280 K	2.2	7.1	89	2.3	7	72
298 K	2.5	6.1	88	2.1	5	74

a Rate constants correspond to *k*_CS_ = 1/τ_2_ and *k*_CR_ = 1/τ_3_ (see text).

b Standard deviations of the time constants are estimated at ±5%.

c VR = relaxation of LES_hot_; CS_hot_ = CS from LES_hot_; CS = charge separation from a relaxed LES; and CR = charge recombination.

The rate constants are calculated as *k*_CS_ = 1/τ_2_ and *k*_CR_ = 1/τ_3_ because in PrCN, there are no apparent contributions of other decay pathways to the time constants for thermalized CS and CR (as opposed to the behavior in Tol, vide infra). This is further justified by the observation that CPET from the relaxed LES is about three orders of magnitude faster than excited-state decay in 9-CN-10-Me-An (τ = 17 ns).^31^ The above results are very close for **1** and **2** in PrCN in our previous study (τ_CS_ = 5.2 ps and 4.6 ps for **1** and **2**, respectively),^31^ but the “hot” species are resolved much better in the present data. Note that the data in the previous study were fitted with two components, and their re-evaluation using three component shows no change to the reported values in DCM or DMF. The small underestimation of τ_CS_ in PrCN in our previous study, however, does not affect its discussion and conclusions.

### Temperature-Dependent Experiments

**Figure 3:**
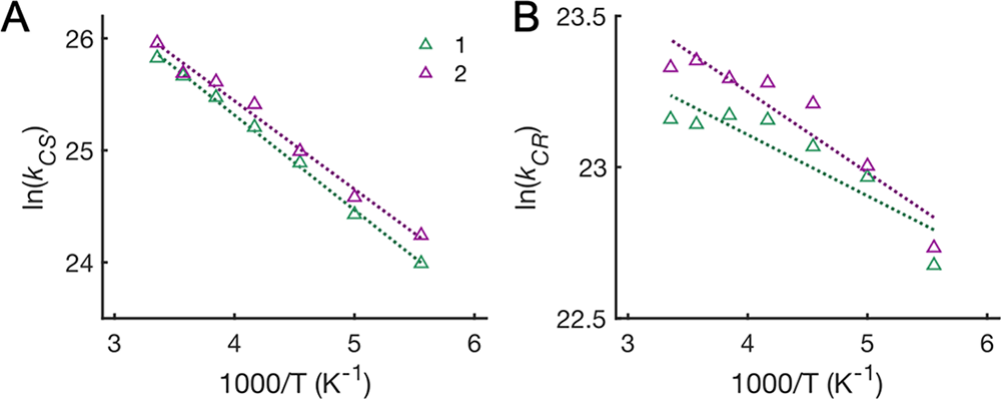
Arrhenius plots (eq 4) for CS (A) and CR (B) for **1** (green) and **2** (purple) in PrCN. The lines correspond to least-squares linear fits. The apparent activation energies are given in the text.

The temperature dependence of CPET CS and CR for **1** and **2** was studied between 298 and 180 K in PrCN, thus avoiding glass formation. Going from 298 to 180 K, the spectral features remain the same. We observe a weak temperature dependence for CS and CR over the 120 K interval examined with ca. sixfold decrease in *k*_CS_ and a mere twofold decrease in *k*_CR_ as the temperature decreases. For both **1** and **2**, the Arrhenius plot (eq 4) shows a good linear relationship for CS (Figure 3A) with an apparent activation energy, *E*_a_, of ∼70 meV (∼1.6 kcal mol^–1^) for both triads. In contrast, the Arrhenius plot for CR is substantially curved (Figure 3B), and linear fits give a crude *E*_a_ estimate of ∼17 meV for **1** (∼0.4 kcal mol^–1^) and ∼ 23 meV for **2** (∼0.5 kcal mol^–1^). *k*_CR_ is essentially constant in the higher end of the studied temperature range, from 240 to 298 K. This is opposite to the expected behavior for a reaction with tunneling activation, where temperature-independent tunneling dominates at low temperatures and the reaction is activated only at higher temperatures. This suggests that the Arrhenius model is, perhaps unsurprisingly, inappropriate to analyze these reactions. Alternative analyses are presented in the Discussion section.
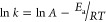
4

### TA Experiments in Nitrile Mixtures

TA experiments were conducted in MeCN/PrCN solvent mixtures to investigate the effect of solvent polarity on the free-energy barriers (ΔG*). The use of MeCN/PrCN solvent mixtures allowed us to systematically vary the static dielectric constant (ε_s_) and refractive index (*n*) from those of pure MeCN (ε_s(293K)_ = 36.64 and *n*_(298K)_ = 1.3414) to those of pure PrCN (ε_s(293K)_ = 24.83 and *n*_(293K)_ = 1.3842).^32^ In addition to having similar specific solute–solvent interactions, nitrile mixtures were selected due to the linear correlation between the solvent mole fraction and dielectric properties as evidenced by the linear shift in absorption λ_max_ of the betaine E_T_(30) probe (see the Supporting Information).

**Figure 4:**
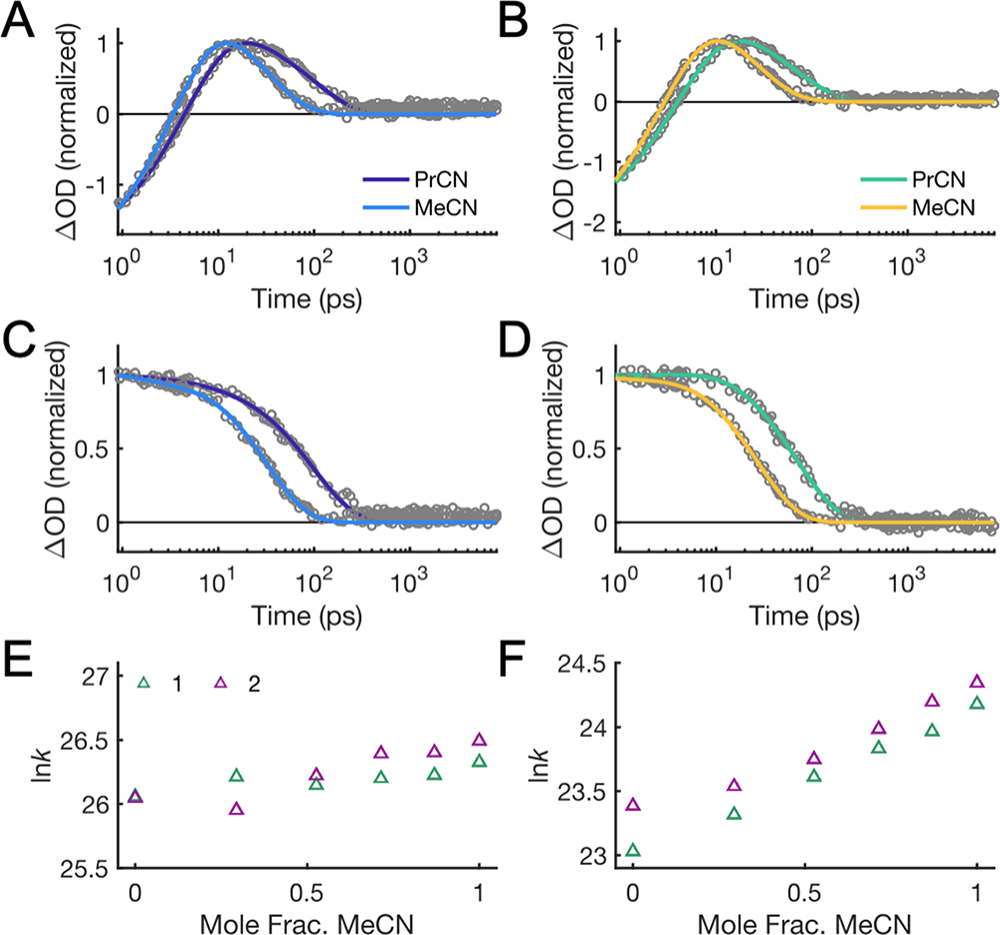
MeCN/PrCN mole fraction dependence of CS and CR kinetics for **1** (A,C) and **2** (B,D) in pure PrCN or MeCN at 293 K. (A–D): Normalized TA time traces (gray circles) with multiexponential fits (colored lines). At 455 nm, the rise and decay of the CSS are observed (A,B), whereas 540 nm is an isosbestic point for LES and CSS, and only decay of the latter is monitored (C,D). (E,F): Rate constants for CS (E) and CR (F) for **1** and **2** as a function of mole fraction of MeCN in the nitrile mixtures.

All the TA spectral features for **1** and **2** in nitrile mixtures are the same as described above at 298 K. For both **1** and **2**, *k*_CS_ and *k*_CR_ systematically increase as the solvent polarity is increased from that of neat PrCN to MeCN (Figure 4, Table 2, and Figures S15–S26). This is illustrated in Figure 4A–D where time traces at a selected wavelength have been chosen to emphasize the kinetic differences in neat solvents for CS and CR. Traces at 455 nm show an initially negative TA signal due to SE from LES_hot_ that is converted to a positive absorption from the CSS and finally followed by decay of the latter to restore the GS. Traces at 540 nm are at an isosbestic point between the LES and CSS and thereby monitor only the CR process. *k*_CS_ shows a modest increase by 30–60% from neat PrCN to neat MeCN. In contrast, *k*_CR_ shows a larger effect with 2.5–3 times larger rates in MeCN than in PrCN for both triads.

**Table 2:** Time Constants^a^*^,^*^b^ and Assignments^c^ from Global Fits to the TA Data in MeCN/PrCN Solvent Mixtures and Their Respective Solvent Parameters

				triad 1			triad 2	
			τ_1_ (ps)	τ_2_ (ps)	τ_3_ (ps)	τ_1_ (ps)	τ_2_ (ps)	τ_3_ (ps)
*X*_MeCN_^d^	ε_s_^e^	*n*^e^	VR + CS_hot_	CS	CR	VR + CS_hot_	CS	CR
1.00	36.64	1.3456	1.6	3.7	32	0.3	3.1	27
0.87	35.10	1.3506	2.3	4.1	39	0.4	3.4	31
0.71	33.27	1.3566	1.8	4.2	45	0.2	3.4	38
0.53	31.05	1.3639	1.2	4.4	56	0.7	4.1	48
0.29	28.30	1.3728	1.5	4.1	75	2.6	5.4	60
0.00	24.83	1.3842	1.0	4.8	100	2.0	4.9	70

a Rate constants correspond to *k*_CS_ = 1/τ_2_ and *k*_CR_ = 1/τ_3_ (see text).

b Standard deviations of the time constants are estimated at ±5%.

c VR = relaxation of LES_hot_; CS_hot_ = CS from LES_hot_; CS = charge separation from a relaxed LES; and CR = charge recombination.

d Mole fraction of MeCN in MeCN/PrCN mixtures.

e Values of ε_s_ and *n* were assumed to vary linearly with the mole fraction.

The observed trend of the dependence of *k*_CS_ and *k*_CR_ on ε_s_ and *n* is consistent with expectations based on the Marcus outer reorganization energies and driving forces (eqs 5−8). In Marcus theory,^11,44^ ΔG* depends on Δ*G*° and λ according to eq 1. The same dependence is found in CPET theory (eq 3), where Δ*G*°_μν_ for each vibronic transition is given. The total reorganization energy for ET and CPET is the sum of inner-sphere (λ_in_) and outer-sphere (λ_out_) contributions (eq 5).
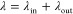
5where λ_in_ is related to changes in bond lengths and angles, while λ_out_ is due to solvent polarization changes between the reactant and product states. By applying a dielectric continuum model and assuming spherical reactants, the following approximation can be made:^11,44^
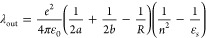
6where *e* is the elementary charge, ε_0_ is the vacuum permittivity, *a* and *b* are the radii of the donor and the acceptor, respectively, and *R* is the distance between their centers. The last parenthetical term in eq 6 describes the dependence of λ_out_ on the solvent ε*_s_* and *n*. λ_out_ is larger in MeCN than in PrCN due to the lower *n* and higher ε_s_ of the former (eq 6). At the same time, the zwitterionic CSS is energetically stabilized as predicted by eqs 7 and 8:
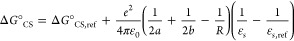
7
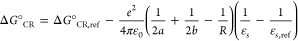
8where the subscript “ref” refers to any reference solvent used for comparison of Δ*G*°.^45^ For CPET reactions like in **1** and **2**, it can be approximated that the charges formed in the CSS reside on the electron acceptor (anthracene) and proton acceptor (pyridine), while phenol remains charge neutral upon oxidation to a phenoxyl radical. Thus, in eqs 6−8, the radii *a* and *b* and the distance *R* should represent those for the anthracene and pyridine couple.

Increasing the MeCN mole fraction in the mixtures increases the dielectric constant and should thereby stabilize the CSS, as given quantitatively in eq 7. For the CS reaction, both the driving force −Δ*G*°_CS_ and λ will then increase, thereby having a counter-balancing effect on ΔG*. This is consistent with the observed small changes in *k*_CS_ for both triads.

For CR on the other hand, increasing the MeCN mole fraction in the mixtures makes Δ*G*°_CR_ less negative (eq 8) while λ increases. This decreases ΔG* and makes the reaction less inverted. Therefore, the large solvent effect observed for CR is consistent with the inverted region behavior since changes in Δ*G*° and λ act in the same direction. A quantitative analysis based on this continuum model is used to investigate the variation of *k*_CS_ and *k*_CR_ in the MeCN/PrCN mixtures (see the Discussion section).

### TA Experiments in Tol

**Figure 5:**
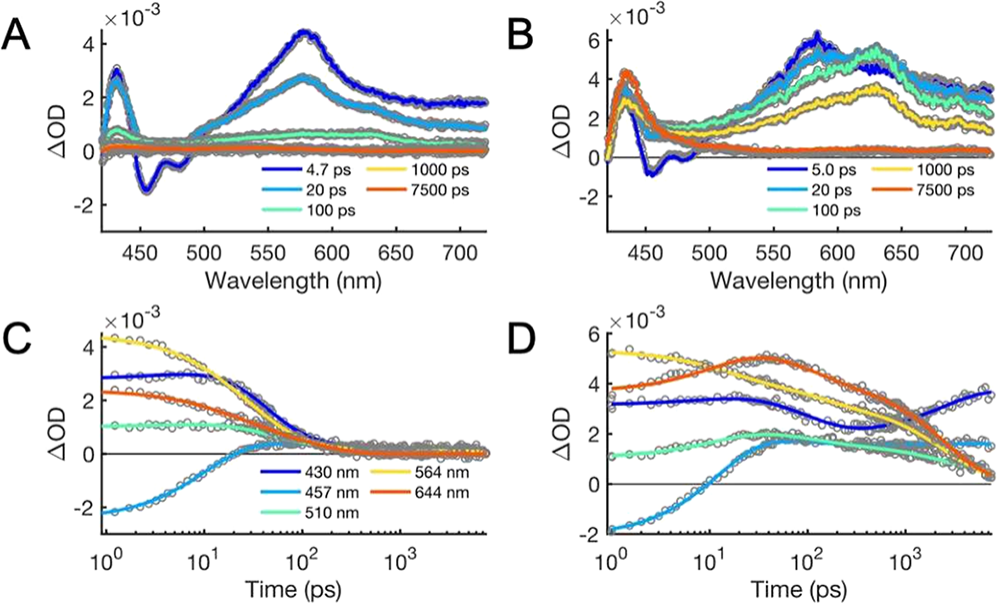
UV–vis TA data of **1** (A,C) and **2** (B,D) in Tol at 298 K after fs excitation at 400 nm. TA spectra (A,B) and time traces at selected wavelengths (C,D). The resulting time constants and assignment of the process are listed in Table 3. Residual plots and normalized EAS from the fits are shown in the Supporting Information.

The CSS should be strongly destabilized in the very low-polarity solvent Tol (ε_s(298K)_ = 2.38) compared to PrCN. Despite its higher energy, the CSS is observed for **1** and **2**, and its assignment can be done as previously described based on the absorption bands at 425 nm (PhO^•^) and at 625 and 670 nm (An^•–^) (Figure 5). Notably, the CSS is more long-lived in Tol than in more polar solvents: τ_CSS_ = 140 ps for **1** and 2.5 ns for **2** at 298 K. The destabilization of the CSS should result in CR driving forces that are higher than those in PrCN (Δ*G*°_CR_(Tol) < −2.48 eV for **1** and < −2.54 eV for **2**), making the observation of a 2.5 ns lifetime remarkable. The vibronic coupling is strong enough to give *k*_CR_ = (27 ps)^−1^ in the polar solvent MeCN. This clear result is a strong demonstration of CPET in the inverted region, where the very low-polarity solvent should result in a low λ.

Another important difference compared to PrCN is a new absorption band at 435 nm, which appears simultaneously with the decay of the CSS and does not decay on the time scale of the TA experiments (τ_4_ ≫ 8 ns). This band has a very small amplitude for **1** but has much stronger amplitude for **2** (Figure 5 and Figure S30). The spectrum for this long-lived component is in good agreement with that of triplet anthracene (^3*^An).^46^

The time constants resulting from the global fit and respective assignments are listed in Table 3. Except for the formation of ^3*^An upon CR, **1** and **2** follow the same reactions described using PrCN. One differentiating factor between **1** and **2** in Tol is that while τ_1_ for **1** represents VR of the initial LES state, CS in **2** seems to be completed during the first component τ_1_ = 12 ps, representing CS_hot_, as determined from the EAS (Figure S30). Therefore, CS is assigned to τ_2_ for **1** and τ_1_ for **2**, while CR is assigned to τ_3_ for both triads. The EAS for both τ_2_ and τ_3_ in **2** show the CSS (bands at 425, 625, and 670 mm). The second component, with τ_2_ = 103 ps, shows only small and spectrally indistinct TA changes, making the EAS of the second and third components very similar. The second component is tentatively assigned to CSS relaxation (although τ_2_ appears to be quite long to support the assignment).

**Table 3:** Time Constants^a^*^,^*^b^ and Assignments^c^*^,^*^d^ from Global Fits to the TA Data in Tol at 298 K

	triad 1			triad 2		
	τ_1_ (ps)	τ_2_ (ps)	τ_3_ (ps)	τ_1_ (ps)	τ_2_ (ps)	τ_3_ (ps)
assignment^c^	VR	CS	CR	CS_hot_	d	CR
	10	32	140	*12*	*(103)*^d^	2541

a Rate constants correspond to *k*_CS_ = 1/τ_2_ and *k*_CR_ = 1/τ_3_ (see text).

b Standard deviations of the time constants are ±5%.

c VR = relaxation of LES_hot_; CS_hot_ = CS from LES_hot_; CS = charge separation from a relaxed LES; and CR = charge recombination.

d For triad **2**, τ_2_ is a minor component, possibly related to CSS relaxation.

Formation yields of the CSS (Φ_CSS_) and ^3*^An (Φ_T_) were estimated relative to the LES, using the EAS amplitudes and extinction coefficients of the species involved (Table 4 and details in the Supporting Information). The Φ_CSS_ values were estimated at ∼26% for **1** and ∼90% for **2**. Assuming similar extinction coefficients in PrCN and Tol, the Φ_CSS_ value for **2** was as high in Tol as in PrCN and within the experimental uncertainty (cf. Figures 4B and 2B). This is in striking contrast to the much lower yield for **1** in Tol versus PrCN (cf. Figures 4A and 2A). The Φ_T_ values were estimated at ∼0.5% for **1** and ∼6.8% for **2**. Thus, the CSS mostly undergoes CR to reform the GS (as in nitrile solvents) with only a minor fraction forming ^3*^An, presumably via intersystem crossing in the CSS state followed by CR to ^3*^An (see the Discussion section).^45,47^

**Table 4:** Formation Yields of ^3*^An and ^1^CSS with Respect to the LES for **1** and **2** in Tol^a^

	^3*^An %		^1^CSS %	
*T* (K)	**1**	**2**	**1**	**2**
298	0.5	6.8	26	90

a Details in the Supporting Information.

## Discussion

Our previous study^31^ allowed the assignment of the LES → CSS conversion in triads **1–3** to a concerted PCET mechanism (CPET) on the basis of the observed kinetic evolution in the TA and thermochemical and kinetic estimates for the different PCET mechanisms. Thus, in a single kinetic step, one *e*^–^ transfers from phenol to the excited anthracene moiety, while H^+^ transfers from phenol to pyridine. This assignment was first based on the simultaneous formation of the spectral features of PhO^•^ and An^•–^ as well as the observed KIE (1.7 ± 0.2), both of which were in agreement with the CPET mechanism. Second, stepwise proton transfer followed by electron transfer (PTET) and electron transfer followed by proton transfer (ETPT) were excluded by considering that the PT step of PTET should be significantly uphill (Δp*K*_a_ > 10, for phenol and pyridinium in MeCN^2^), which would not allow for the observed rate constant of ∼10^11^ s^–1^. The initial ET seemed inefficient, emphasized by the use of a reference triad where the phenolic hydroxyl group was replaced by a methoxy group, which did not show any quenching of ^1^*An.^42^ Finally, the free-energy dependence of the observed *k*_CS_ for the CSS formation in **1**–**8** varied with Δ*G*°_CPET_, in agreement with theoretical predictions for CPET (eq 3) but not with ETPT or PTET.

In PrCN, the CSS spectroscopic features of PhO^•^ and An^•–^ showed simultaneous and monotonic decay to the GS, thus closing a simple three-state photochemical cycle.^31^ An analysis of this CR based on thermochemical and kinetic estimates for the different PCET mechanisms, analogous to that for CS, allowed us to assign the mechanism to CPET. As described in the Introduction, the CPET CR displayed an inverted region behavior, both in a comparison of **1–3** in the same solvent and for each compound when the solvent polarity was varied. The prior computational modeling suggested that vibronic transitions around 0 → 3 dominate this reaction for triad **1** in DCM with an activation barrier ΔG*_0,3_ = 90 meV. A table of the relative contribution of the most important vibronic transitions and their parameters is provided in the Supporting Information (Table S2).

In this section, we will analyze and discuss the reaction barriers for CS and CR, using the data for the MeCN/PrCN mixtures and the temperature-dependent data in PrCN, and provide further experimental evidence of the inverted region character of CR. In particular, we explore whether the explanation for the inverted region behavior, such as being dominated by transitions to higher vibrational product states (the 0 → 3 vibronic transition when in DCM),^31^ is supported by experimental estimates of the effective ΔG* and −Δ*G*° values. The latter values should in turn reflect a weighted average of the vibronic transitions contributing to each reaction. Thereafter, we will discuss the results in the nonpolar solvent Tol, which show that the CR can indeed be pushed further into the inverted region to give nanosecond lifetimes of the CSS.

### Marcus-Type Modeling—Motivation for Fits with One Averaged Transition

As a first approximation to model the solvent polarity and temperature dependencies of CPET, we use a Marcus-type fit (eq 2) for the nitrile mixture and temperature-dependent data in PrCN. An analysis using eq 3 is not feasible here as it contains too many unknown parameters to allow for a fit to the kinetic data. The Marcus-type fit represents a gross simplification for a CPET reaction as eq 3 includes additional temperature-dependent factors beyond the classical barrier ΔG* for the 0,0 transition. First, the Boltzmann population of proton vibrational states of the reactant (*P*_μ_) can give a temperature dependence. For the present CR reactions, however, computational results showed that only transitions from μ = 0 were important because of the relatively high energy of μ = 1 and above.^31^ Second, the distribution of PT distances (*R*_PT_) increases in width with increasing temperature, and this is expected to add to the experimentally observed activation energy. Calculations indicate that the dominant proton donor–acceptor distance is close to the equilibrium distance, thereby suggesting a minimal temperature dependence of *R*_PT_. Third, because multiple vibronic transitions are predicted to contribute to the rate, each with a different ΔG*_μν_, the observed effective ΔG* is a weighted average of these transitions. In an effort to assess the latter effect, we have adapted the previous computational results for CR in DCM (Table S2)^31^ to PrCN by adding 60 meV to Δ*G*°_μ,ν_ in the more polar PrCN and using a value of λ = 1.3 eV in PrCN (Table 5, see reasoning below and the Supporting Information for details on the value for λ). In Table 5, it is assumed that all CSS energies are 60 meV lower than those in the less polar solvent DCM, relative to the GS, but that the proton vibrational wavefunction overlaps (*S*^2^_μ,ν_) remain unchanged. Table 5 therefore shows which vibronic transitions contribute most to *k*_CR_ and gives the values of their respective energetic and overlap factors.

**Table 5:** Computational Data for the Vibronic Transitions That Are Predicted To Give the Main Contributions to the CR Rate Constant for **1** in PrCN at 298 K, cf. Eq 3^a^

(μ,v)	*P*_μ_	Δ*G*°_μv_	ΔG*_μv_	*S*^2^_μv_	exp	% contrib.^b^
0–0	1.00	–2.48	0.27	1.03 × 10^–3^	3.18 × 10^–5^	0.00
0–1	1.00	–2.26	0.18	4.53 × 10^–1^	9.49 × 10^–4^	9.63
0–2	1.00	–2.20	0.16	4.24 × 10^–1^	2.23 × 10^–3^	21.19
0–3	1.00	–2.06	0.11	1.03 × 10^–1^	1.39 × 10^–2^	31.95
0–4	1.00	–1.90	0.07	1.73 × 10^–2^	7.02 × 10^–2^	27.21
0–5	1.00	–1.71	0.03	1.45 × 10^–3^	2.83 × 10^–1^	9.20
0–6	1.00	–1.51	0.01	4.99 × 10^–5^	7.28 × 10^–1^	0.81
0–7	1.00	–1.28	0.00	4.78 × 10^–8^	9.98 × 10^–1^	0.00
0–8	1.00	–1.05	0.01	1.41 × 10^–7^	6.24 × 10^–1^	0.00

a Values adapted from ref (31), Table S2, for DCM by adding 60 meV to Δ*G*°_μv_ to account for the solvent changes (eqs 7 and 8) and using λ = 1.3 eV (see the Supporting Information). The vibrational wavefunction integrals as well as the relative energy differences between the Δ*G*°_μv_ values are assumed to be the same as reported for DCM.

b Relative contribution (%) to the overall *k*_CPET_ calculated from the products of the preceding two columns.

The following sections show how a qualitative and even semi-quantitative agreement with the previous theoretical modeling can be obtained from a semi-classical Marcus-type fit of the temperature dependence of *k*_CR_ in which the manifold of vibronic transitions in eq 3 is represented by a weighted average according to eq 2, yielding an averaged value of Δ*G*°_CR_ from the fits. This value is compared with Δ*G*°_μv_ of the calculated vibronic transitions that are predicted to give the largest contribution to *k*_CR_ (Table 5). The effective ΔG* and Δ*G*° values obtained from the fits to the temperature-dependent and MeCN/PrCN data are in good agreement with each other and correspond to the computationally predicted most important transitions based on the values in Table 5.

### CPET Reactions in MeCN/PrCN Solvent Mixtures

Changing the mole fraction of MeCN in the MeCN/PrCN mixtures changes Δ*G*° and λ, thereby changing ΔG* according to eq 1, in a Marcus-type analysis. To investigate whether the observed changes in *k*_CS_ and *k*_CR_ with the mole fraction of MeCN followed the predicted dependence of eqs 1 and 2, our data were fitted according to eq 9a that is obtained by rearranging eq 2. In eq 2, the pre-exponential factor (equals *B* in eq 9b) contains the solvent-dependent term . Multiplying both sides of eq 2 by  and rewriting in a logarithmic form gives eq 9a, where the factor *C* is independent of the dielectric properties of the solvent.
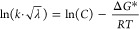
9a
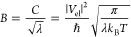
9b

**Figure 6:**
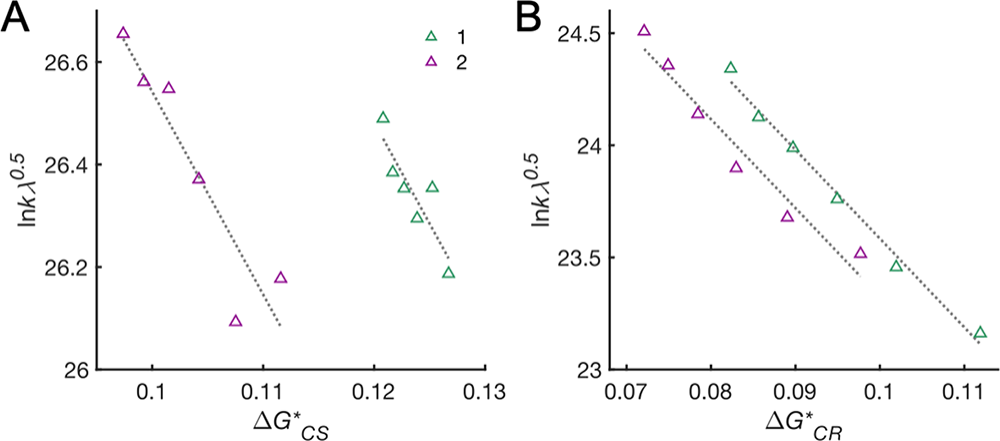
Kinetic data and fits according to eq 9a for CS (A) and CR (B) in **1** and **2** in MeCN/PrCN mixtures. λ_PrCN_ = 1.3 eV for both reactions (see Table 6).

The data of  plotted versus ΔG* should give a straight line with a slope equal to −(*RT*)^−1^ (*T* = 293 K). The values of ΔG* for each solvent composition were calculated from the values of Δ*G*° and λ in PrCN and their estimated variation with the solvent. The variation in λ was estimated using the expressions for charged spheres in a continuum (eqs 5 and 6), assuming that ε_s_ and *n* vary linearly with the mole fraction of the solvents. The driving forces for CS and CR were also assumed to vary linearly with the solvent mole fraction. The difference in Δ*G*° for CS and CR between the neat solvents was previously calculated to be 60 meV,^31^ which is in fair agreement with predictions from eqs 7 and 8 (30 meV). In ref (31), −Δ*G*°_CS_ in PrCN was calculated to be ∼0.49 eV and ∼0.54 eV for **1** and **2**, respectively. A value of λ, similarly estimated by calculations, was used; see next paragraph. The CS data were fitted by using these predetermined Δ*G*°_CS_ and λ values in PrCN as input parameters for the analysis. Eqs 5 and 6 were then used to calculate λ in MeCN, as described above, and the value of Δ*G*°_CS_ in MeCN was varied until a good fit with a slope equal to −(*RT*)^−1^ was obtained. The CR data were fitted independently following the same procedure, with the same value of λ as for CS. The resulting fits according to eq 9a were good, as shown in Figure 6. Note that CR and CS are expected to have very similar λ values considering the small structural rearrangements between the GS and LES of An, as inferred by the small Stokes shift of the 0–0 lines in the absorption and fluorescence spectra (Figure 2).

For the choice of λ used in the fits, we used the value of λ = 1.4 eV previously calculated in DCM for **1** as a reference for which λ_in_ = 0.38 eV (cf. eq 5).^31^ From eqs 5 and 6, we can expect a value of λ = 1.5 eV in the more polar solvent PrCN. The results for CS with λ_PrCN_ = 1.5 eV however gave unphysically large pre-exponential factors (∼1 × 10^14^ s^–1^; Table 6), whereas a value of λ_PrCN_ = 1.3 eV gave quite reasonable results. The latter value also agreed with the results of the temperature-dependent analysis, see below, and was therefore preferred. The difference in Δ*G*°_CS_ between the neat solvents for **1** (70 meV) was in good agreement with the previous calculations above, while the difference was slightly larger (110 meV) for **2**. The more negative Δ*G*°_CS_ in MeCN was counterbalanced by a larger λ, such that there was ≤30% variation in *k*_CS_ between the two neat solvents. The pre-exponential factor obtained (*C* in eq 9a) was reasonable and corresponds to a Marcus pre-exponential factor in eq 2 of *B* = (2 ± 1) × 10^13^ s^–1^ for **1** and **2** (Table 6).

**Table 6:** Resulting Parameters for the Neat Solvents from a Fit According to Eq 9a to the Kinetic Data in MeCN/PrCN Mixtures

triad	λ/eV	solvent	Δ*G*°_CS_/eV	ΔG*_CS_/eV	*B*_CS_/s^–1^	Δ*G*°_CR_/eV	ΔG*_CR_/eV	*B*_CR_/s^–1^
1	1.3	PrCN	–0.49	0.13	3.1 × 10^13^	–2.06	0.11	8.0 × 10^11^
		MeCN	–0.56	0.12		–2.06	0.08	
	1.5	PrCN	–0.49	0.17	1.7 × 10^14^	–2.17	0.07	1.8 × 10^11^
		MeCN	–0.57	0.16		–2.14	0.05	
2	1.3	PrCN	–0.54	0.11	1.5 × 10^13^	–2.01	0.10	6.1 × 10^11^
		MeCN	–0.65	0.10		–2.01	0.07	
	1.5	PrCN	–0.54	0.15	8.0 × 10^13^	–2.12	0.06	1.6 × 10^11^
		MeCN	–0.66	0.14		–2.09	0.04	

In PrCN, the Δ*G*°_CR_ value for the 0 → 0 transition was calculated to −2.48 and −2.43 eV for **1** and **2**, respectively.^31^ A fit according to eq 9a required even more negative values in MeCN: Δ*G*°_CR_ = −2.51 and −2.53 eV for **1** and **2**. These results were unreasonable as the more polar MeCN should further stabilize the CSS compared to PrCN. In addition, this would imply that the combined equilibrium driving forces for CS and CR (−Δ*G*°_CS_, −Δ*G*°_CR_) would exceed the excited-state energy (*E*_00_ = 2.97 eV) of the LES. Hence, the results seem to indicate an overestimation of −Δ*G*°_μν_ for the CR and its most contributing vibronic transitions. Instead, Table 5 suggests that the transitions giving the main contributions to the rate constant in PrCN are centered around the 0 → 3 transition, which has a much lower driving force than the 0,0 transition: Δ*G*°_0,3_ = −2.06 eV. Acceptable linear fits for CR with λ_PrCN_ = 1.3 eV, resulting in a slope of −(*RT*)^−1^, were in fact obtained with Δ*G*°_CR:PrCN_ ≈ −2.06 eV for **1** and ∼ −2.01 for **2** (Figure 6 and see the Supporting Information for discussion of fits). This supports our previous computational results, suggesting CR to higher vibrational states with a CR thermal barrier (ΔG*). It should be noted that the averaged ΔG*_CR_ is smaller for **2** than **1**, which can be explained by the somewhat lower energy of the CSS in **2**, making the CR less inverted. The difference in ΔG*_CR_ between the dyads is, however, still smaller than the difference in ΔG*_CS_, which is consistent with the general notion of a shallower driving force dependence of the rate constant in the inverted region.

The difference in effective Δ*G*°_CR_ between neat PrCN and MeCN is negligible, which can be understood from the fact that as the CSS is stabilized in the more polar solvent, transitions to lower vibrational states become more important. Table S4, constructed on the basis of Table 5, highlights the different vibronic contributions in the nitrile mixtures, where Δ*G*°_CR: MeCN_ is near that of the 0 → 2 transition (see the Supporting Information for description). It is evident however that the reason for the considerable change in ΔG*_CR_ and observed *k*_CR_ between solvents (ranging from DCM to DMF in ref (31)) cannot be traced back to solely changes in Δ*G*°_CR_ but rather the significant change in λ.^31^

To conclude, the nitrile mixture rate constants can be satisfactorily modeled with eq 9a with values of λ and Δ*G*° that are close to those calculated for λ and Δ*G*°_μν_ of the dominating vibronic transitions. For CR, Δ*G*°_CR_ is clearly less negative than for the 0 → 0 transition, and by comparison with calculations from ref (31), it represents an effective average around the 0 → 3 transitions in the nitrile mixtures. The much stronger increase in the CR rates with increased mole fraction of MeCN, compared to the CS, is consistent with a combined effect of an increase in λ and a less negative Δ*G*°, making the reaction less inverted. This observation and the fit results support the assignment of the CR occurring in the inverted region with a significant effective barrier of ΔG* ∼0.1 eV in PrCN, as computed in ref (31).

### CEPT Temperature Dependence in PrCN

Rate constants for CS and CR are smaller for **1** than **2** over the temperature interval examined (Table 1). This is consistent with the ca. 50 meV higher energy of the CSS for **1** that results in a smaller driving force in the normal region CS and a larger driving force in the inverted region CR.

Despite a temperature change by almost 120 K, from 180 to 298 K, the variation in the CS and CR rate constants for both **1** and **2** in PrCN is small (Table 1). A previous study of the CS reaction for the parent triad **6**, using fluorescence measurements, found a very small temperature dependence for CS between 145 and 350 K in 2-methyltetrahydrofuran (Me-THF) with an Arrhenius activation barrier of only ∼14 meV (0.33 kcal mol^–1^).^42^ A similar result may be expected for CR in the inverted region, based on prior observations for ET systems. ET reactions in the inverted region are strongly affected by nuclear tunneling to excited vibrational states of the products.^13,14^ This not only decreases the classically expected falloff of rate with further increase of −Δ*G*° but it may also be expected to result in a weak temperature dependence, as observed experimentally.^34,35^

**Figure 7:**
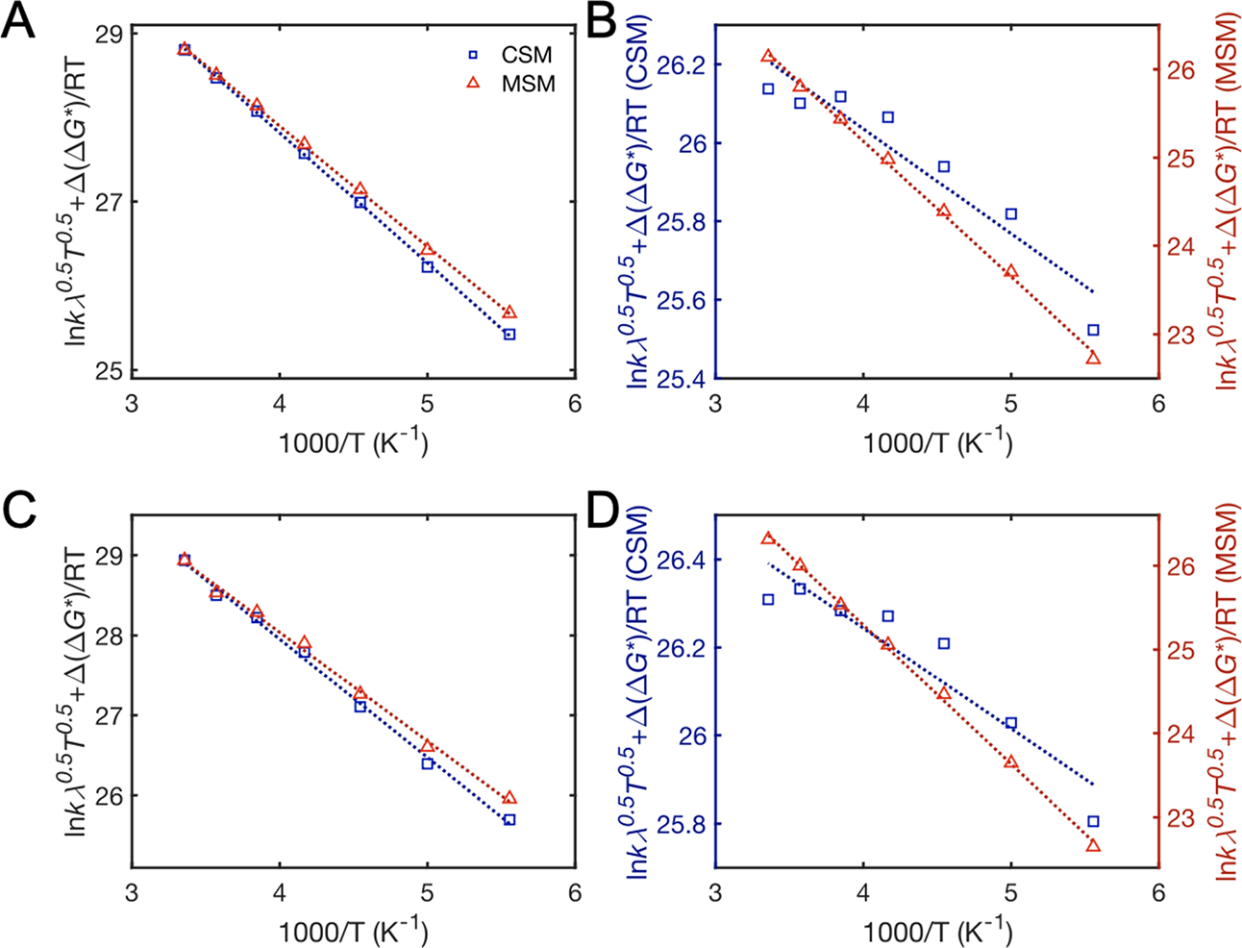
Data and fits for CS (A,C) and CR (B,D) for **1** (A,B) and **2** (C,D). The red and blue lines are fit according to eq 10, accounting for the temperature dependence of Δ*G*° and λ using either CSM (blue) or the results of MSM (red). The MSM model predicts a decrease in λ with increasing temperature, in contradiction with continuum models but in analogy with ref (55); see text. λ_298K_ = 1.3 eV was used for the plots.

Classical and semi-classical Marcus expressions provide a foundation to analyze the temperature dependence of ET reactions (cf. eq 2). Typically, a plot of ln *k*_ET_ versus 1/*T*, or ln *k*_ET_·*T*^1/2^ versus 1/*T* (to account for the weak temperature dependence of the pre-exponential factor), is assumed to yield a straight line with a slope equal to −ΔG*/R. In a corresponding analysis of our CPET data, we find that ln *k*_CS_ correlates linearly with 1/*T* (cf. Figure 3). In contrast, neither ln *k*_CR_ nor ln *k*_CR_·*T*^1/2^ versus 1/*T* yields linear correlations, and a clear downward curvature is observed instead (Figure 7).

The above approach may be justified over a small temperature range, which is suitable when ΔG* is large. For large ranges of temperature, the variation of Δ*G*° and λ with temperature may be significant, which is attributed to the reaction entropy and reorganization entropy, respectively.^48−50^ The CSS is a zwitterionic species that polarizes the solvent, resulting in a smaller entropy than for the charge neutral LES and GS. Corrections for the temperature dependence of Δ*G*° and λ in ET reactions have in many cases been made by using the temperature dependence of *n* and ε_s_ and the continuum models of eqs 5−8.^34,35,51−54^

The dielectric properties of PrCN change substantially upon cooling from 298 to 180 K: *n* increases from 1.38 to 1.44 and ε_s_ increases from 24.3 to 43.6 (see the Supporting Information for details). The much larger solvent polarity at lower *T* stabilizes the CSS. However, according to eq 6, λ also decreases as *T* decreases because of the larger effect from the concomitant increase in *n*. The net result is that ΔG* for CR is predicted to increase as *T* is lowered. This is in contrast to the results from the studies of, for example, Liang et al.^34^ and Serpa et al.^54^ in nonpolar solvents Me-THF and isopropyl ether, respectively. In these solvents, eq 6 predicts that λ increases as *T* decreases because the increase of ε_s_ is more important than that of *n*.

Here, the predicted changes of ΔG* with temperature were calculated from eqs 5−8 and the temperature-dependent data for ε_s_ and *n* (see the Supporting Information). To obtain a linear fit of ln *k* versus 1/*T*, a term was added in the fit, representing the change in barrier  as the temperature was lowered from 298 to 180 K. In order to also correct for the small changes due to (*T*·λ)^−1/2^ in the pre-exponential factor, the kinetic data were therefore plotted versus 1/*T* according to eq 10:
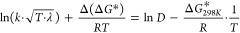
10where *D* encompasses the terms of the pre-exponential factor from eq 2 (). The initial input values of λ and Δ*G*° at 298 K for an iterative optimization, guided by the calculations of Table 5, were used to find a satisfactory linear fit to the data, with a resulting slope (−Δ*G*_298K_^*^/*R*). Here, the iterative process of fitting according to eq 10 aimed to find input values of λ_298K_ and Δ*G*°_298K_ that gave a value of ΔG*_298K_ according to eq 1 that was identical to the value of ΔG*_298K_ returned by the fit.

Satisfactory fits of the CS data were obtained with eq 10, but the CR data have a clear downward curvature (Figure 7). The downward curvature cannot be attributed to the failure of the implicit assumption of a single, averaged vibronic transition. Parameters of a full CPET model, which were neglected when assuming a single transition (cf. eqs 3 and 2; see discussion above), become more important with increasing temperature. In turn this would rather cause an upward curvature of the plots. This is verified by a simple summation of the rate contributions from the main vibronic transitions shown in Table 5 at different temperatures (Table S7). Instead, the downward curvature of the data plotted according to eq 10 (Figure 7, blue data points) suggests that the continuum solvent expressions in eqs 5−8 fail to properly correct the temperature dependence of Δ*G*°_CR_ and λ.

Shortcomings of the continuum model (eqs 7 and 8) to accurately describe the temperature dependence of Δ*G*° and λ have been discussed by several authors.^55−64^ In the molecular solvent models (MSMs) developed by Matyushov et al.^55^ and Newton et al.,^60^ solvent density fluctuations are important components of λ_out_ but are obviously neglected in dielectric continuum solvent models (CSMs). In a study of particular relevance for the present work, Matyushov et al. applied MSM to interpret the temperature dependence of the CR of a donor–acceptor molecule in MeCN.^55^ The study found that the predicted decrease of λ with increasing temperature reproduced the experimental temperature dependence, while the continuum model prediction of increase of λ with increasing temperature failed to do so.

We believe that the situation is similar in the present system in which we study the CR of the CSS back to a charge neutral GS in a nitrile solvent. A full theoretical calculation according to the molecular model is far beyond the scope of the present study. Instead, we attempted a fit according to eq 10 but now calculating Δ(ΔG*) with the assumption that λ decreased linearly with increasing temperature in a similar way as in ref (55). The magnitude of the temperature dependencies (slopes) Δλ/Δ*T* and Δ(Δ*G*^°^)/Δ*T* will obviously depend on the details of the donor–acceptor molecule studied and will presumably be somewhat different for MeCN used in ref (55) and PrCN used here. We employed several different slopes of Δ*G*° and λ, using those of ref (55) as the starting point. Ultimately, we only considered slopes that yielded values of Δ*G*°_CS_, Δ*G*°_CR_, λ and the pre-exponential factor (*B*) at 298 K, which are physically reasonable. Thus, Δ*G*°_CS_ and λ were selected to be within 50 meV and 0.3 eV, respectively, of the calculated values from ref (31), with −(Δ*G*°_CS_ + Δ*G*°_CR_) ≤ *E*_0,0_ and the pre-exponential factor *B* ≤ 3 × 10^13^ s^–1^ (Table S9).

Rewardingly, this procedure yields good linear correlations (*R*^2^ ≥ 0.99) with a combination of physically reasonable parameters for only a rather small range of slopes. The best fits have the same relative slopes of −Δ*G*° and λ versus *T* as those obtained in ref (55), with Δ*G*°_CR_ and λ decreasing by ∼100 and ∼50 meV, respectively, from 180 to 298 K, and Δ*G*°_CS_ increasing (becoming less negative) by ∼100 meV. These variations with temperature can be compared with the predictions from eqs 7 and 8, where Δ*G*°_CR_ and λ both change by ∼50 meV but in opposite directions. The room-temperature parameter values obtained from the best fits for **1** and **2** are given in Table 7.

**Table 7:** Fit Results for **1** and **2** in PrCN at 298 K Using Eq 10 and MSM^a^

triad	*T*/K	λ/eV	Δ*G*°_CS_/eV	ΔG*_CS_/eV	*B*_CS_/s^–1^	Δ*G*°_CR_/eV	ΔG*_CR_/eV	*B*_CR_/s^–1^
1	298	1.30	–0.49	0.13	2.4 × 10^13^	–2.15	0.14	5.1 × 10^12^
2	298	1.30	–0.52	0.12	2.0 × 10^13^	–2.19	0.15	8.8 × 10^12^

a The range of values for acceptable fits (see text) are: ±0.05 eV (Δ*G*°_CS_), ±0.2 eV (Δ*G*°_CR_), ±0.01 eV (ΔG*_CS_), ±0.04 eV (ΔG*_CR_), ±0.1 (λ), ±1.0 (B_CS_), and ± 2.0 (*B*_CR_), respectively; see the Supporting Information for details.

The results are in good agreement with the fits to the data from MeCN/PrCN mixtures at 298 K (Table 6) and are consistent with the calculations shown in Table 5. In particular, the CR evidently occurs predominantly in higher vibronic states of the electronic GS. The effective Δ*G*°_CR_ obtained is very similar to that of the calculated 0 → 3 transition (−2.06 eV) as shown in Table 5, which is the same effective Δ*G*°_CR_ used in the fit of the MeCN/PrCN mixtures. The value of ΔG*_CR_ = 0.14 ± 0.04 eV for **1** is in good agreement with the value of 0.11 eV from the fit to the MeCN/PrCN mixture data. Thus, we believe that the fitting model (eq 10 with a MSM correction), although simplified, is semi-quantitatively correct. The narrow parameter range of acceptable fits to the temperature-dependent rate constants is in good agreement with the fits of data from the MeCN/PrCN mixtures, and the corresponding calculated data shown in Table 5 support that notion.

The differences between the MSM and CSM models are less significant for the MeCN/PrCN mixture data. Although we used a mole fraction-averaged Pekar factor () in eq 6 to calculate λ and ΔG*, the errors are presumably similar for the two nitrile solvents. It is also little doubtful that MeCN, with a larger *n* and smaller ε_s_ than PrCN, gives both a larger λ and a more stabilized CSS. Therefore, we believe that the actual variation in Δ(ΔG*) in these solvent mixtures is similar to the one predicted in our fit to eq 9a.

The fits for the MSM give a much stronger variation in  with temperature than the CSM (Figure 7B,D). This is because ΔG*_CR_ is predicted to be much larger with the MSM than with the CSM (e.g., for **1**: ∼0.14 vs 0.023 eV at 298 K). On the other hand, the variation of  for the CS step is very similar for the MSM and CSM fits, which may seem surprising given the fact that λ changes with temperature in opposite directions for the two models. The reason for the similar result is that ΔG*_CS_ is predicted to be very similar and shows quite parallel changes with temperature for the two models. With decreasing temperature, λ increases by ca. 40 meV in the MSM instead of decreasing by a similar amount in the CSM, but this is compensated by the fact that Δ*G*°_CS_ decreases twice as much in the MSM. Thus, the term Δ*G*°_CS_ + λ of the numerator in eq 1 is the same within ∼30 meV in the two models. The greater difference between MSM and CSM for CR than for CS is not because the former is in the inverted region but because of the opposite dependencies of Δ*G*°_CS_ and Δ*G*°_CR_ on temperature.

To conclude, variable temperature experiments in PrCN confirm our previous observation of inverted region CR and are consistent with calculations using the PCET theory. The effective barrier for the CR, determined from the fits, is ΔG*_298K_ = 0.14 ± 0.04 eV, which is similar to the barrier previously suggested by calculations of the dominating vibronic transitions (Table 5).^31^ The value is also similar to that obtained in the fits to the data in MeCN/PrCN mixtures at 298 K, ΔG*_CR_ = 0.11 eV for **1**. This supports our claim that CR occurs in the inverted region and that it has a distinct barrier for the classical coordinates, instead of occurring entirely via activationless nuclear tunneling.

### CEPT in Toluene

The differences in the interplay between Δ*G*° and λ in the normal and inverted regions become larger in nonpolar solvents. In analogy to previous discussion, in Tol, we observe the expected qualitative trends in CS and CR, that is, in Tol, a decrease of both −Δ*G*° and λ is expected for CS (maintaining a counter-balancing effect on ΔG*), whereas an increase in −Δ*G*° and a decrease in λ is expected for CR, thereby pushing the reaction further into the inverted region. In agreement with these trends, there are larger differences in *k*_CS_ versus *k*_CR_ in Tol compared to the more polar solvents (cf. Tables 3 and 2, respectively). (*k*_CS_)^−1^ is on the order of 10–30 ps in Tol, similar to the values observed in PrCN and MeCN, while CR becomes significantly slower, so that the CSS decays back to the GS with τ ∼ 2.5 ns for **2**.

Reliable estimates of Δ*G*° and λ for CS and CR in nonpolar solvents are typically challenging.^65−69^ Estimates of λ_out_ become negligibly small because 1/ε_s_ and 1/*n*^2^ become equally large (eq 6). Estimates of Δ*G*° using eqs 7 and 8 predict that the CSS is destabilized by ∼0.9 eV in Tol compared to PrCN; this is obviously exaggerated as it would make the CS endergonic by ∼0.4 eV. Similar cases of rapid ET (ps time scale) in Tol have been reported before but where continuum models predict endergonic CS.^65−69^ Here, we can only conclude that Δ*G*°_CS_ in Tol must be somewhere between −0.06 eV (because the conversion of LES to CSS is at least 90% complete) and −0.49 eV in the stabilizing polar solvent PrCN. Estimates of λ_out_ using eqs 5 and 6 would predict a value of ∼0 eV and therefore λ ≈ λ_in_ ≈ 0.38 eV (see above). The MSM predict a larger λ_out_ in nonpolar solvents; however, typically λ_out_ = 0.1–0.3 eV,^70^ suggesting a value of λ = 0.5–0.7 eV. Thus, it seems that λ > −ΔG°_CS_ in our case, that is, CS is in the normal region. Assuming that the pre-exponential factor for CS in **1**, where the 0 → 0 transition would dominate, it would be similar in Tol and PrCN (*B* = 3 × 10^13^ s^–1^, Table 6), and the observed *k*_CS:Tol_ = 1/τ_2_ ≈ 3 × 10^10^ s^–1^ (Table 3) would be consistent with a barrier of ΔG*_CS_ ≈ 0.15 eV in Tol at room temperature. This estimate is in agreement with the suggested range of values above, for example, Δ*G*°_CS:Tol_ = −0.06 eV and λ = 0.7 eV, which would produce a barrier of ca. 0.15 eV (eq 1). These estimates point to a CSS energy for **1** in Tol around 0.1–0.2 eV below the singlet LES (2.97 eV), that is, at ∼2.8 eV relative to the GS.

Destabilization of the CSS, and the resulting long lifetime of the CSS, allows for the formation of a long-lived ^3*^An state as the CSS recombines. ^3*^An lies ca. 1.75 eV above the GS,^71^ such that Δ*G*°_CR_ to ^3*^An should be ∼ −1.0 eV. With the estimated value of λ = 0.6–0.9 eV, this reaction should be in the inverted region, but less inverted than the singlet CR (Δ*G*°_CR_ ∼ −2.8 eV). Nevertheless, the quantum yield for ^3*^An formation is low, ∼ 7% for **2** and even smaller for **1**, ∼0.5%. However, for the latter, the ^3*^An extinction coefficient at the absorption maximum (435 nm) is an order of magnitude larger than that for the CSS allowing for its observation in the TA spectra (Figure S30). Formation of ^3*^An during CR requires that the CSS first undergoes intersystem crossing (ISC), that is, singlet–triplet conversion, most likely via hyperfine interactions as described for related radical ion pairs.^46,47,72,73^

ISC in radical ion pairs of organic molecules typically occurs on the time scale of >1 ns,^46^ such that it can only compete with CR of the ^1^CSS when its lifetime approaches this time scale like in Tol for **1** and **2**. A similar feature with a narrow 435 nm band at long delay times was observed in the low-polarity solvent DCM employed in our previous study where *k*_CR_*=* (755 ps)^−1^.^31^ ISC should be slow on the time scale of CR and limit the yield of ^3*^An formation. Indeed, the low yield of ^3*^An shows that direct CR to the GS dominates the observed CR process, with the triplet pathway giving only a minor contribution to the rate.

The CSS formation yield of **2** in Tol, and of both triads in PrCN, is Φ_CSS_ ≥ 90% based on literature extinction coefficients (see the Results section and the Supporting Information). The much lower Φ_CSS_ for 1 in Tol (∼26%) cannot be explained by direct deactivation to the GS as the LES lifetime of cyanoanthracene is three orders of magnitude longer than that in **1**. This suggests that there is another parallel deactivation process in **1** in Tol, which is not active in **2** to the same extent and cannot be identified by the present TA experiments. We note that Sayfutyarova et al.^74^ have suggested, based on a computational study, direct formation of a local electron–proton transfer state, with a phenoxyl–pyridiniumyl biradicaloid subunit (An-PhO^•^-pyH^•^). Its potential experimental verification would require different experiments that are beyond the scope of the present study.

## Conclusions

In this study, the photoinduced PCET CS and CR rates of triads **1** and **2** were studied as a function of temperature and solvent polarity. Corrections for the temperature dependence of the reorganization energy and driving force, and the consequent change in the activation barrier with temperature, were attempted with both CSM and MSM.^55^ Satisfactory fits to the data could be obtained using a simplified model with a single vibronic transition (cf. eq 2) that represents a weighted average of the contributing vibronic transitions (cf. eq 3) and using MSM to correct for the temperature dependence of the activation barrier. The fit results suggested that CR had an effective (averaged) barrier of ΔG*_298K_ = 0.14 ± 0.04 eV and a reaction free energy of Δ*G*°_CR,298K_ ≈ −2.1 eV for **1**. The latter value is much less negative than that for CR between the lowest proton vibrational states of CSS and GS (μ = ν = 0). The results match the predictions based on previous calculations^31^ that transitions to proton vibrationally excited states of the electronic GS (0 → 2 and 0 → 3 in PrCN) dominate the CR reactivity. Consistent fits were obtained for the room-temperature data in the PrCN/MeCN mixtures. Here, the contributing vibronic transitions are slightly lower (0 → 1, 0 → 2, and 0 → 3) because the more polar MeCN stabilizes the CSS. Nonetheless, the CR still forms a hot GS with a similar effective ΔG* as in the temperature study.

Both analyses of the temperature and nitrile mixture experiments resulted in pre-exponential factors for CS close to the theoretical limit of ≲1 × 10^13^ s^–1^ (from transition-state theory or dynamic fluorescence Stokes shift^75,76^). The value for Δ*G*°_CS_ from the fit was equal to that for the 0 → 0 transition. This is consistent with a reaction in the normal region, with a moderate barrier of ΔG* ∼0.1 eV, for which the proton wavefunction overlap is good (*S*_μν_^2^ not much smaller than unity) and does not strongly limit the pre-exponential factor. For CR, the pre-exponential factor is 1–1.5 orders of magnitude smaller than that for CS. This is consistent with the computational results shown in Table 5 that transitions to higher proton vibrational states, for which ΔG*_μν_ is small, are limited by a small wavefunction overlap and the transitions with the best wavefunction overlap have a higher ΔG*_μν_ value. Thus, the effective average corresponds to a transition with a moderate barrier of ΔG* ∼0.14 eV and a wavefunction overlap around *S*_μν_^2^ ∼0.1, which are approximately the values for the 0 → 3 transition. We note that the actual barrier is thus larger than that suggested by the weak temperature dependence of the experimental CR rate constant, which is a consequence of the concomitant variation in barrier with temperature.

The results support previous assignment of CR to a concerted PCET reaction in the inverted region.^31^ The model explains why an inverted region behavior is possible, thanks to a poor proton wavefunction overlap for the barrierless transitions. Computational studies have suggested that conditions for the inverted region behavior of CPET are dependent on asymmetric, double well proton potentials, while more symmetric potentials would not give an inverted region behavior.^33^ It is interesting to note that most computational studies of CPET oxidations of small-molecule phenol–base systems suggest highly asymmetric proton potentials.^25,26,33^ Nevertheless, an inverted region behavior has only been reported for the present series of triads. As discussed before,^4^ there are very few studies that report even a curvature in the rate versus free-energy correlation, as is suggested by eqs 2 and 3.^77,78^ It seems that the relation between proton potentials and rate versus free-energy dependence is not yet fully understood on a combined theoretical and experimental level.

In the nonpolar solvent Tol, CR occurs much more slowly with a time constant of ∼2.5 ns for **2** at 298 K. This is a clear manifestation of the inverted region, where the expected decrease in λ and a more negative Δ*G*°_CR_ lead to an even larger reaction barrier. The very slow CR in Tol leads to distinctly different excited-state dynamics with other deactivation pathways competing with the inverted CR. These pathways include spin conversion/PCET to form the spectrally identified long-lived triplet anthracene state.

Overall, this report confirms and enriches our understanding of the PCET reactivity of two triads that undergo CR in the MIR. The results of this study have implications for future developments of reactions relying on the utilization of high-energy, proton-coupled CS states such as photoredox catalysis and solar fuel technologies.

## Supporting Information Available

Additional TA data and fit results and details of the analysis and fits to temperature- and solvent polarity-dependent data (PDF)
