# Supplementary material for: Solvent and Temperature Effects on Photoinduced Proton-Coupled Electron Transfer in the Marcus Inverted Region
Source: J Phys Chem A. 2021 Aug 25;125(35):7670–84. doi: 10.1021/acs.jpca.1c05764 (PMC8436208; doi:10.1021/acs.jpca.1c05764)
Supplement: Supplementary file 1 — jp1c05764_si_001.pdf [file jp1c05764_si_001.pdf]

Supplementary Information:

Solvent and Temperature Effects on Photoinduced Proton-Coupled Electron Transfer in the Marcus Inverted Region.

Laura F. Cotter[1†], Belinda Pettersson Rimgard[2†], Giovanny A. Parada[1,†,§], James M. Mayer[1]*, Leif Hammarström[1]*.

[1] Yale University, Department of Chemistry, New Haven, Connecticut, 06520, USA
[2] Uppsala University, Department of Chemistry – Ångström Laboratory, Uppsala University, Box 523, SE75120 Uppsala, Sweden

[†]equally contributing authors

§ Present address: The College of New Jersey, Department of Chemistry, Ewing, New Jersey 08628, USA

*Correspondence to: leif.hammarstrom@kemi.uu.se, james.mayer@yale.edu

Table of Contents

1. Femtosecond UV-Vis Transient Absorption Spectroscopy 2
   1.1. Temperature Dependence of Triad **1** in PrCN 2
   1.2. Temperature Dependence of Triad **2** in PrCN 5
   1.3. Triad **1** in MeCN and PrCN Mixtures 8
   1.4. Triad **2** in MeCN and PrCN Mixtures 11
   1.5. Triads **1** and **2** in Toluene, 298 K 14
      1.5.1 Formation yields of the ^{1}CSS and ^{3*}An and Target Analysis. 16
2. $E_T(30)$ Measurements for Butyronitrile and Acetonitrile Mixtures 17
3. Solvent and Temperature Dependent Analysis 19
   3.1. Marcus-Type Analysis: MeCN/PrCN Solvent Mixture 19
      3.1.1. Vibronic Contributions to the Charge Recombination: DCM and MeCN/PrCN mixture 20
   3.2. Marcus-Type Analysis: Temperature Study 22
      3.2.1. Vibronic Contributions to the Charge Recombination: Temperature dependence 24
      3.2.2. Matyushov Model – Linearizing the temperature dependence. 25

1. Femtosecond UV-Vis Transient Absorption Spectroscopy

1.1. Temperature Dependence of Triad **1** in PrCN

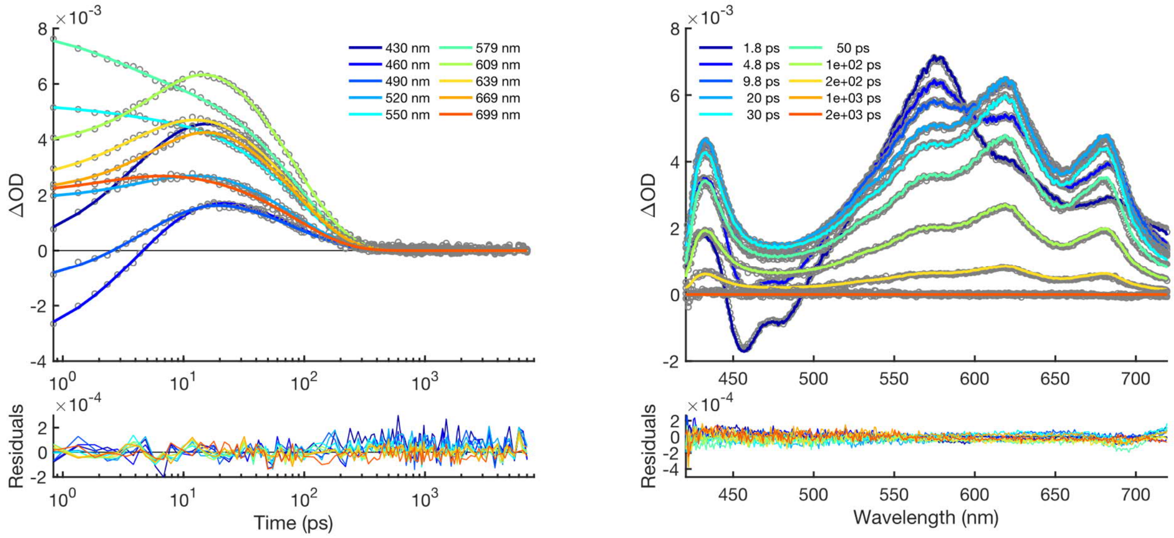

Figure S1. UV-vis TA spectroscopy of **1** in PrCN at 298K at selected wavelengths (left) and selected times (right). Time constants obtained from global analysis are τ_1 = 2.5 ps, τ_2 = 6.1 ps, and τ_3 = 88 ps.

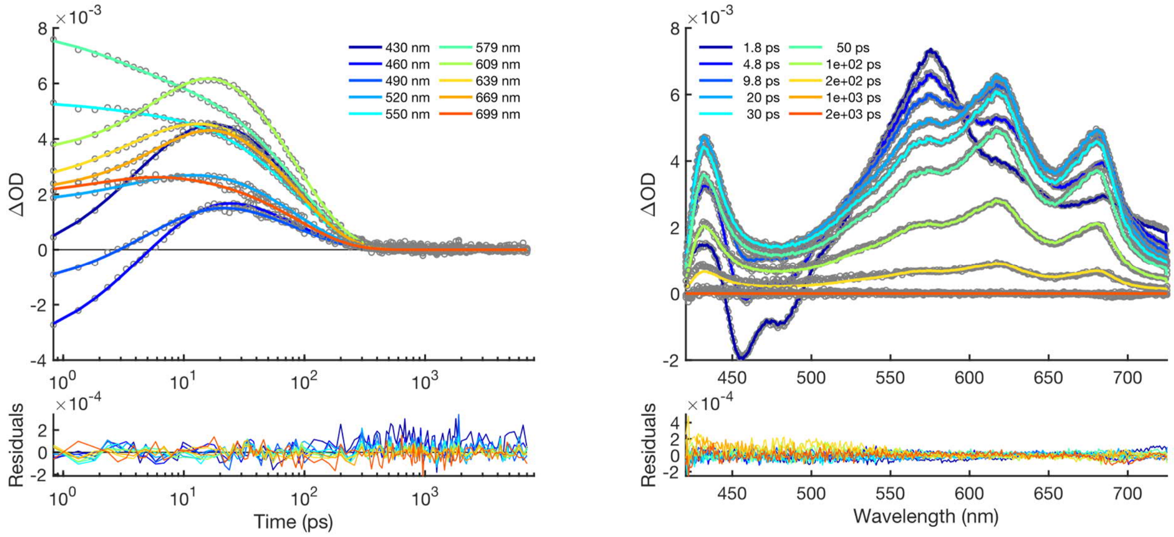

Figure S2. UV-vis TA spectroscopy of **1** in PrCN at 280K at selected wavelengths (left) and

selected times (right). Time constants obtained from global analysis are τ_1 =2.2 ps, τ_2 = 7.1 ps, and τ_3 = 89 ps.

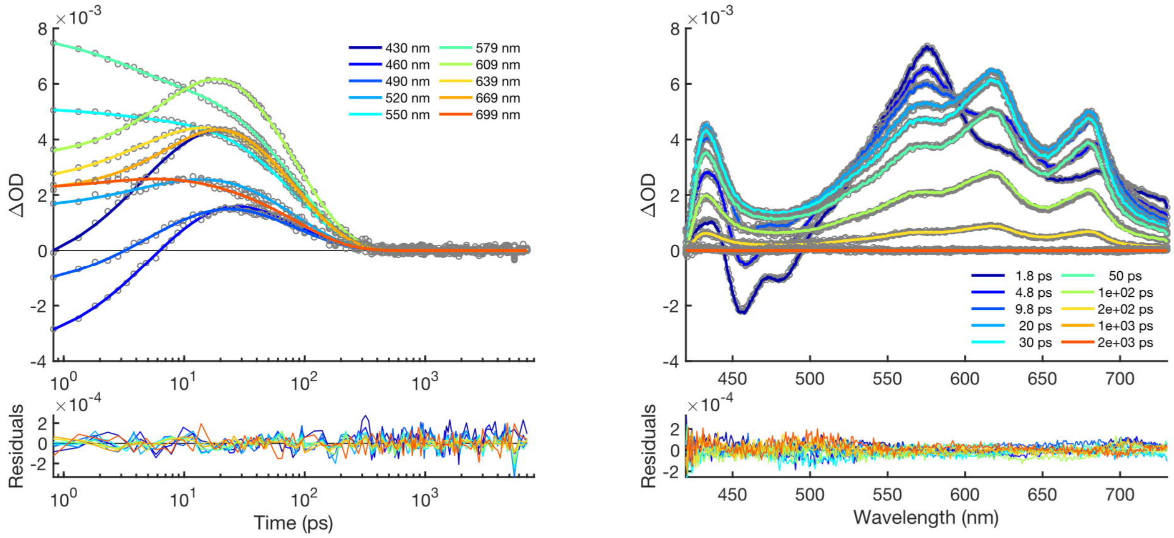

Figure S3. UV-vis TA spectroscopy of **1** in PrCN at 260K at selected wavelengths (left) and selected times (right). Time constants obtained from global analysis are τ_1 = 2.9 ps, τ_2 = 8.7 ps, and τ_3 = 86 ps.

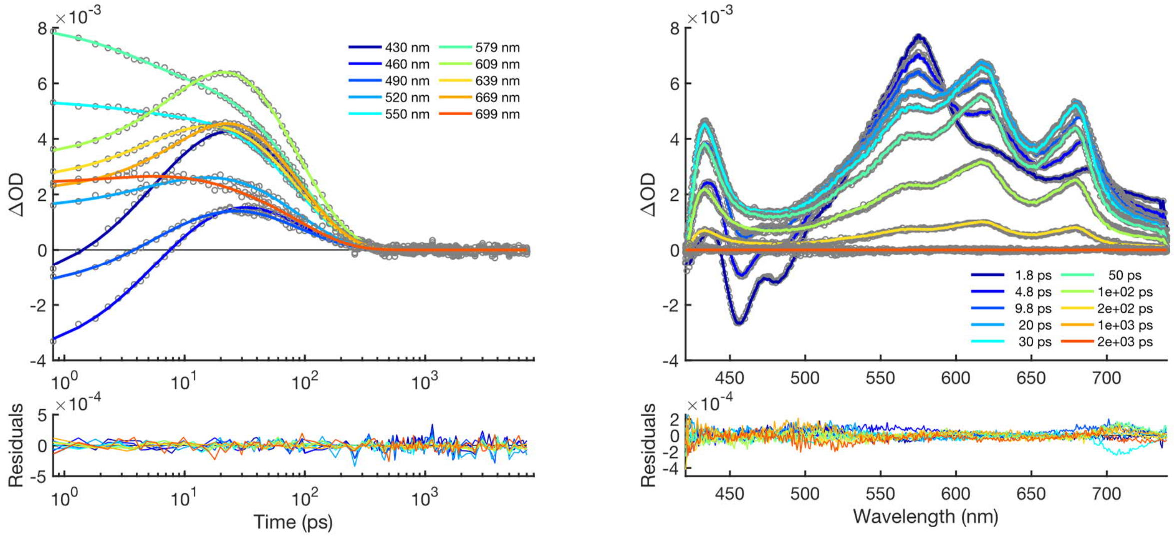

Figure S4. UV-vis TA spectroscopy of **1** in PrCN at 240K at selected wavelengths (left) and selected times (right). Time constants obtained from global analysis are τ_1 = 3.8 ps, τ_2 = 11 ps, and τ_3 = 88 ps.

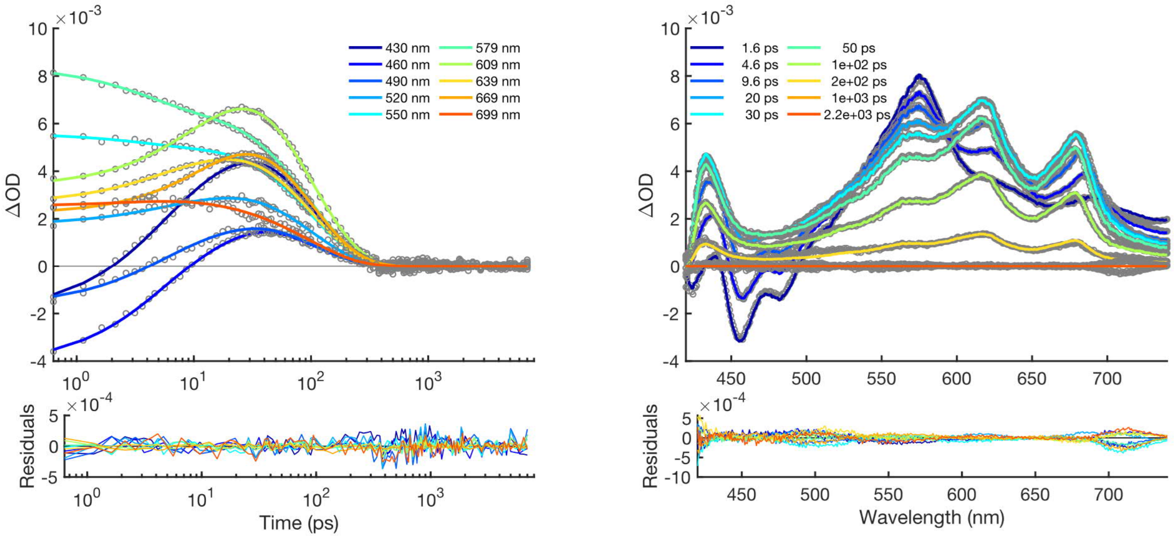

Figure S5. UV-vis TA spectroscopy of **1** in PrCN at 220K at selected wavelengths (left) and selected times (right). Time constants obtained from global analysis are τ_1 = 3.8 ps, τ_2 = 16 ps, and τ_3 = 96 ps.

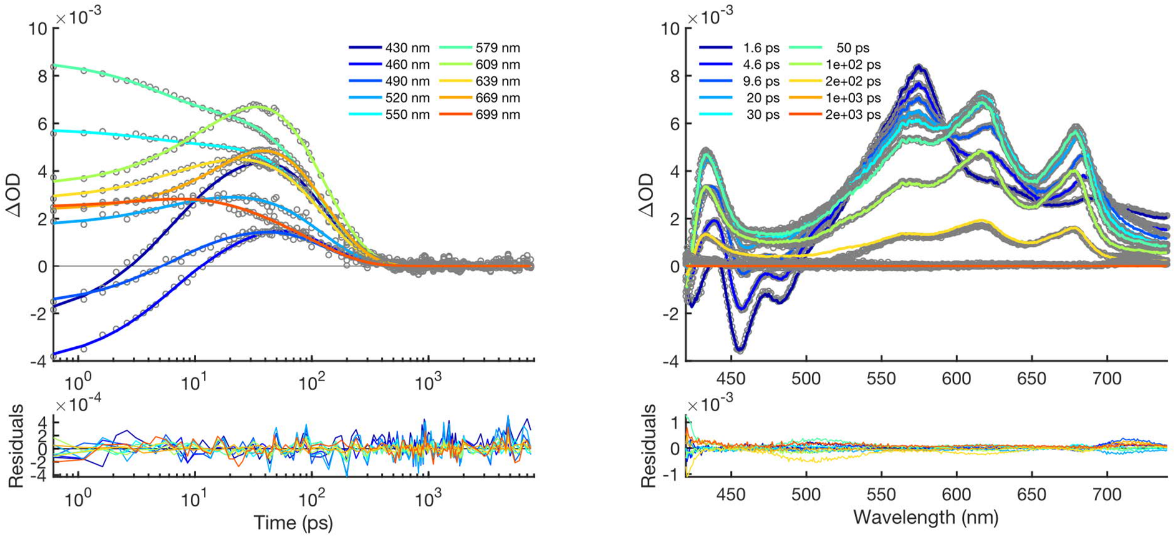

Figure S6. UV-vis TA spectroscopy of **1** in PrCN at 200K at selected wavelengths (left) and selected times (right). Time constants obtained from global analysis are τ_1 = 4.9 ps, τ_2 = 25 ps, and τ_3 = 106 ps.

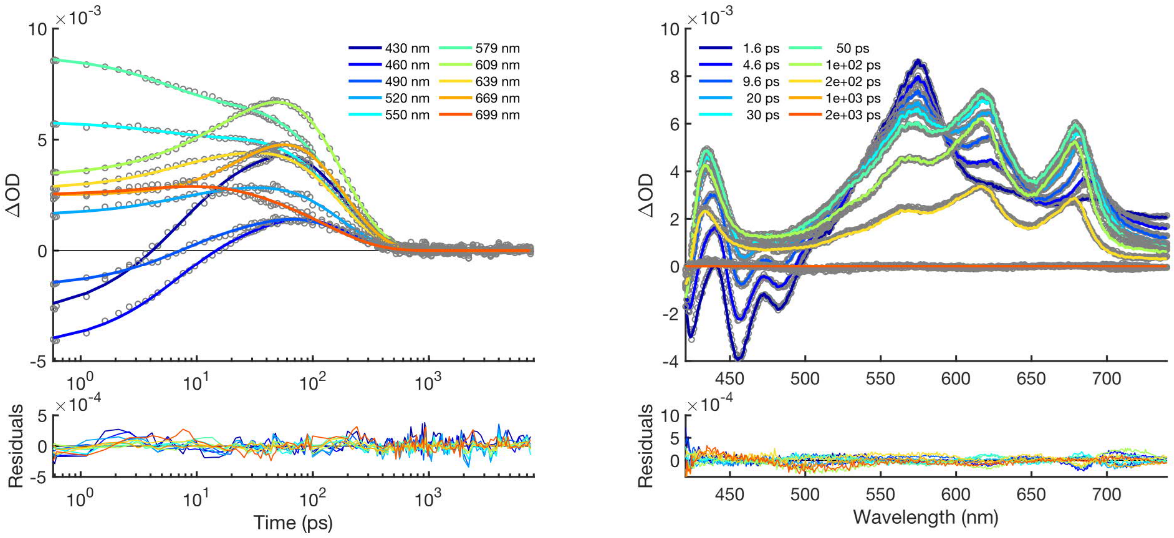

Figure S7. UV-vis TA spectroscopy of **1** in PrCN at 180K at selected wavelengths (left) and selected times (right). Time constants obtained from global analysis are τ_1 = 5.6 ps, τ_2 = 38 ps, and τ_3 = 142 ps.

1.2. Temperature Dependence of Triad **2** in PrCN

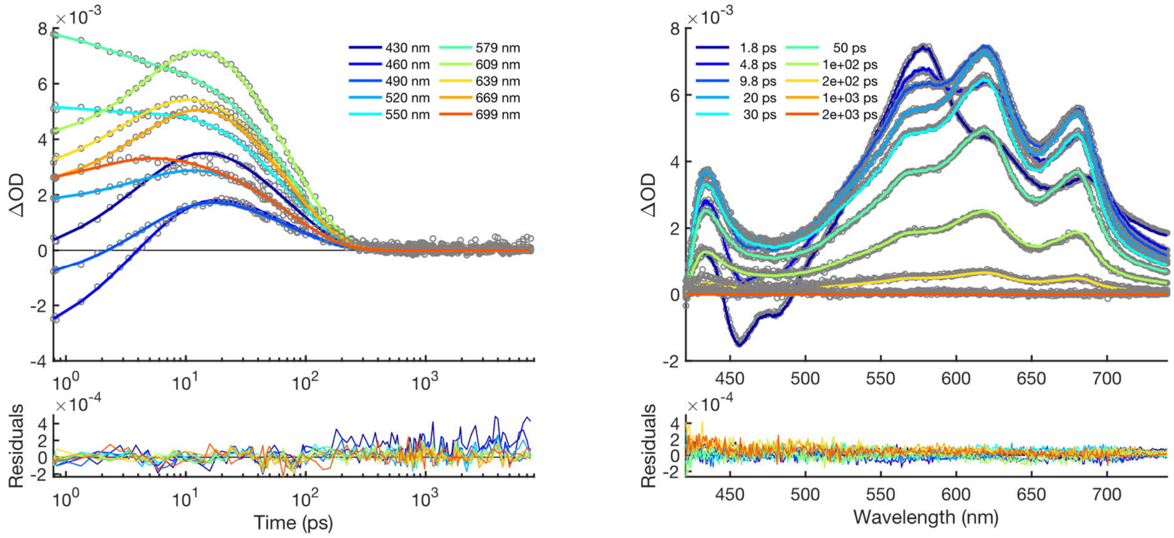

Figure S8. UV-vis TA spectroscopy of **2** in PrCN at 298K at selected wavelengths (left) and selected times (right). Time constants obtained from global analysis are τ_1 = 2.1 ps, τ_2 = 5.3 ps, and τ_3 = 74 ps.

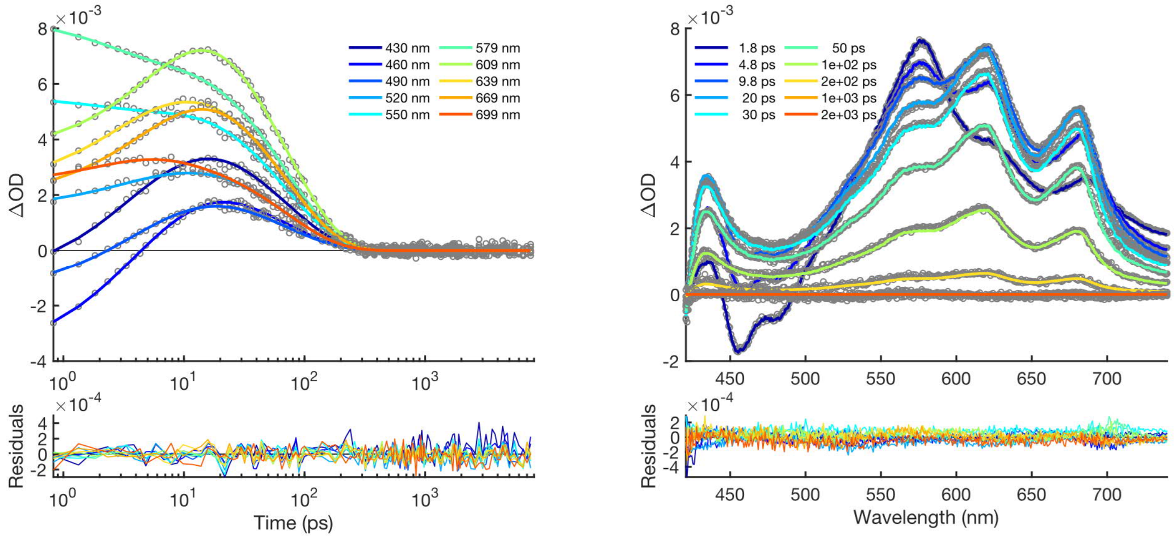

Figure S9. UV-vis TA spectroscopy of **2** in PrCN at 280K at selected wavelengths (left) and selected times (right). Time constants obtained from global analysis are τ_1 = 2.3 ps, τ_2 = 7.0 ps, and τ_3 = 72 ps.

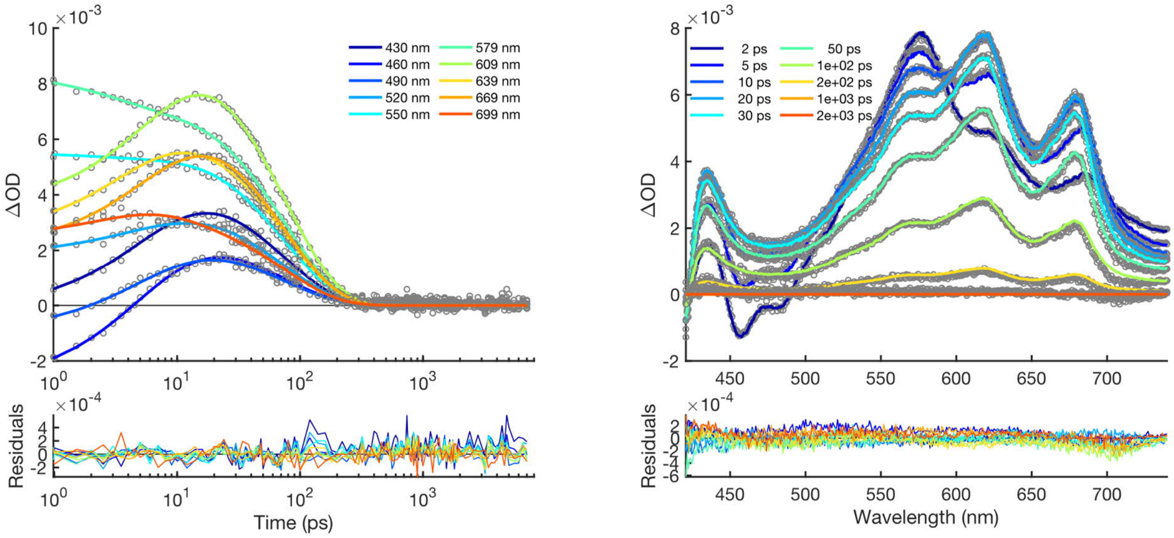

Figure S10. UV-vis TA spectroscopy of **2** in PrCN at 260K at selected wavelengths (left) and selected times (right). Time constants obtained from global analysis are τ_1 = 3.2 ps, τ_2 = 7.5 ps, and τ_3 = 77 ps.

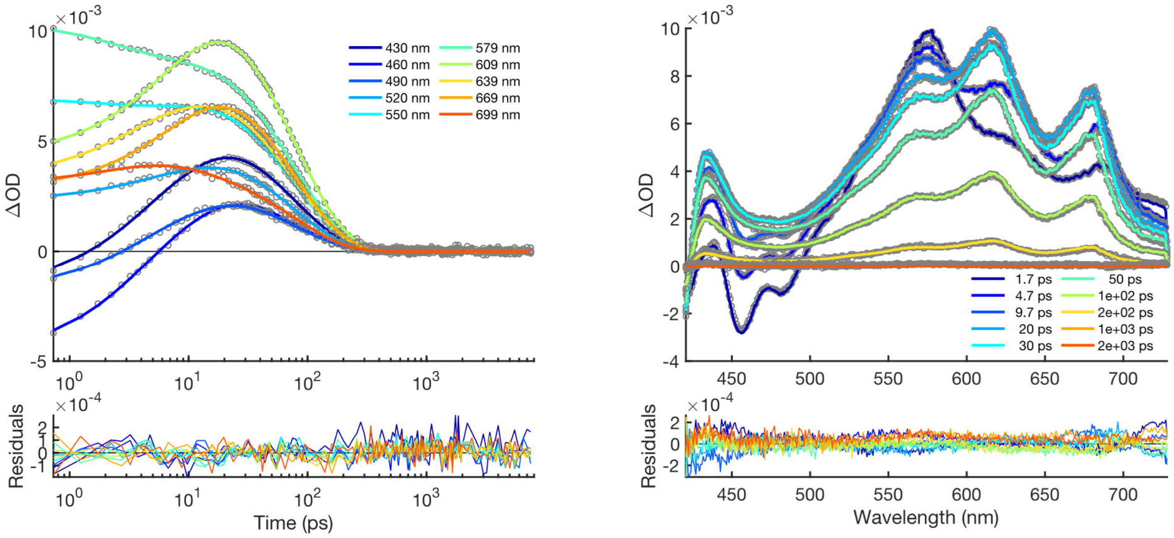

Figure S11. UV-vis TA spectroscopy of **2** in PrCN at 240K at selected wavelengths (left) and selected times (right). Time constants obtained from global analysis are τ_1 = 2.7 ps, τ_2 = 9.2 ps, and τ_3 = 78 ps.

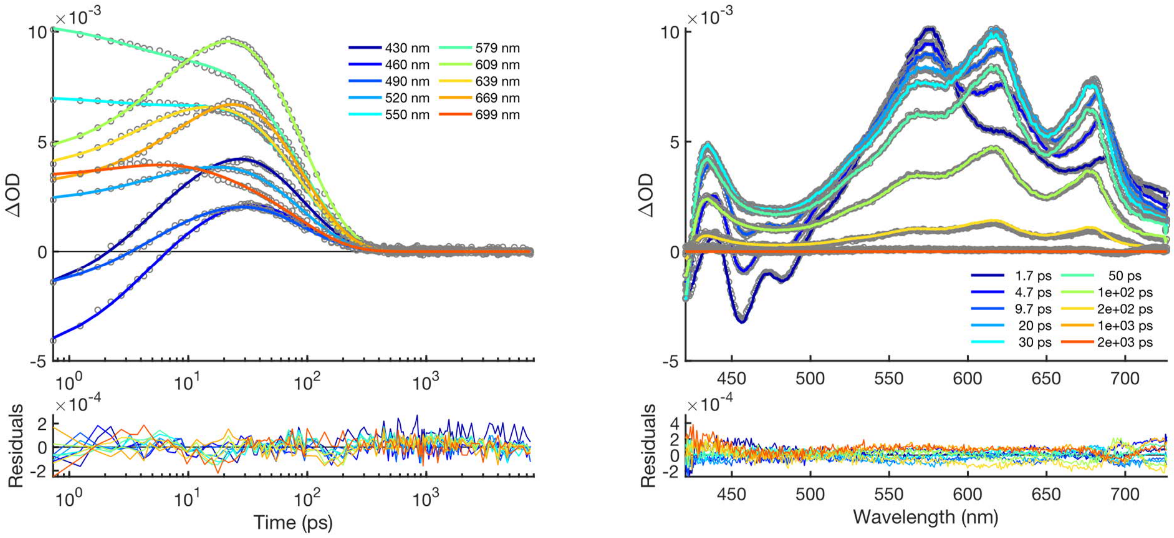

Figure S12. UV-vis TA spectroscopy of **2** in PrCN at 220K at selected wavelengths (left) and selected times (right). Time constants obtained from global analysis are τ_1 = 3.3 ps, τ_2 = 14 ps, and τ_3 = 83 ps.

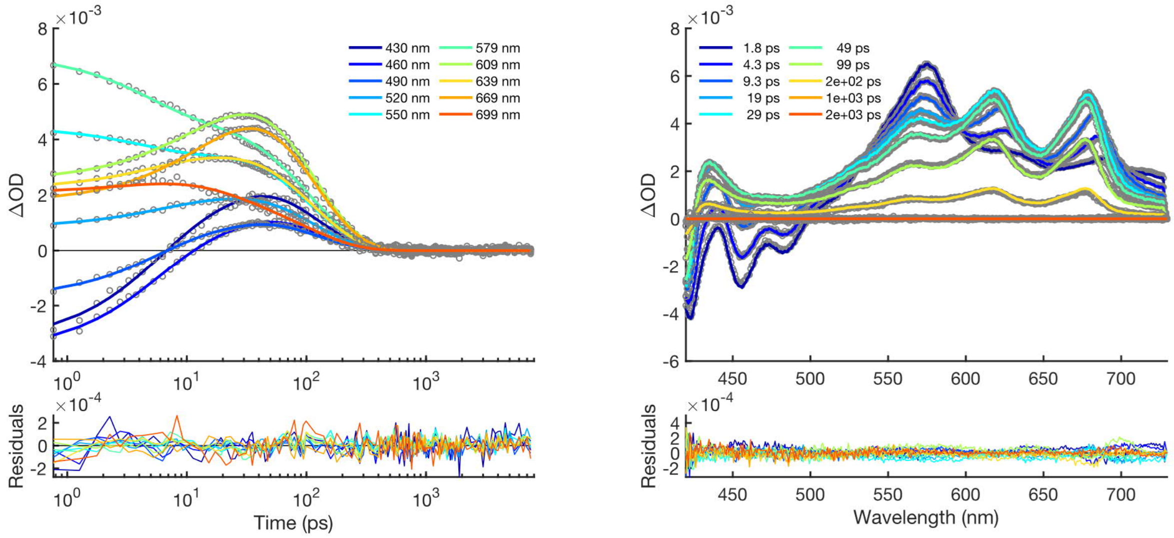

Figure S13. UV-vis TA spectroscopy of **2** in PrCN at 200K at selected wavelengths (left) and selected times (right). Time constants obtained from global analysis are τ_1 = 4.4 ps, τ_2 = 21 ps, and τ_3 = 102 ps.

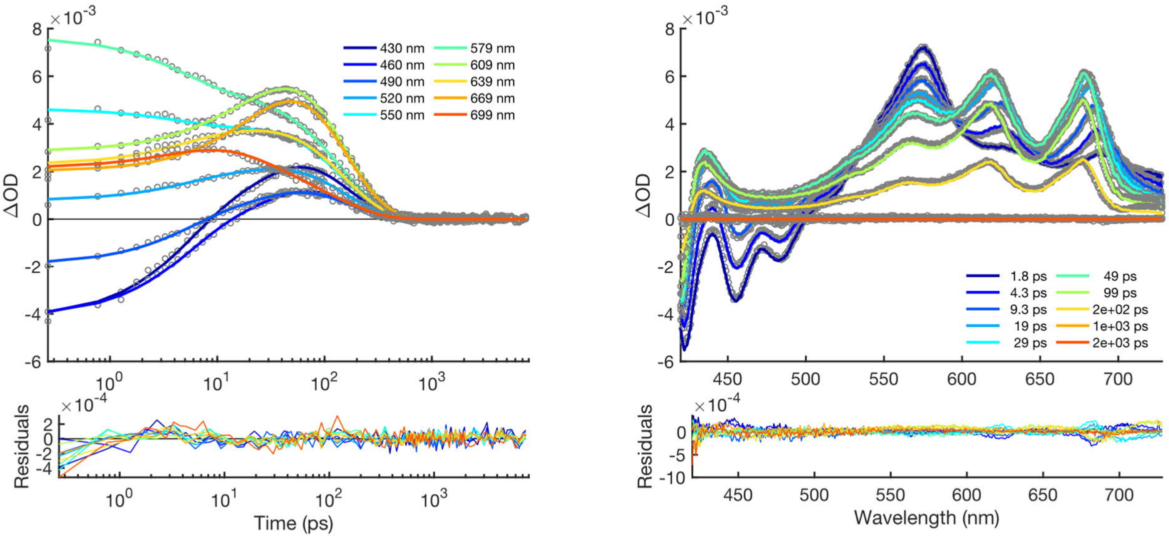

Figure S14. UV-vis TA spectroscopy of **2** in PrCN at 180K at selected wavelengths (left) and selected times (right). Time constants obtained from global analysis are τ_1 = 4.7 ps, τ_2 = 30 ps, and τ_3 = 134 ps.

1.3. Triad **1** in MeCN and PrCN Mixtures

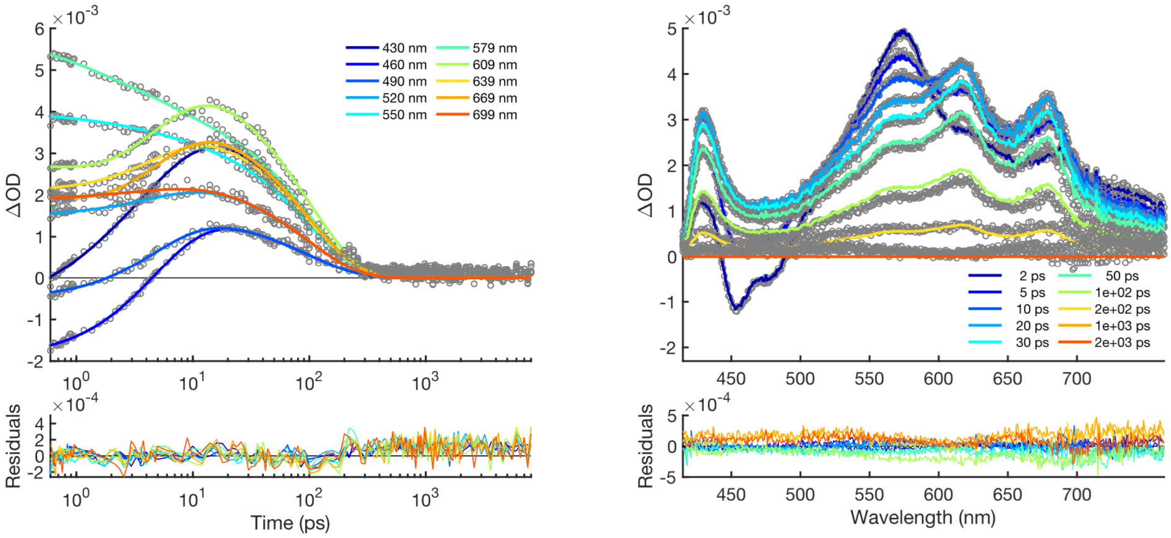

Figure S15. UV-vis TA spectroscopy of **1** in pure PrCN at selected wavelengths (left) and selected times (right). Time constants obtained from global analysis are τ_1 = 1.0 ps, τ_2 = 4.8 ps, and τ_3 = 100 ps.

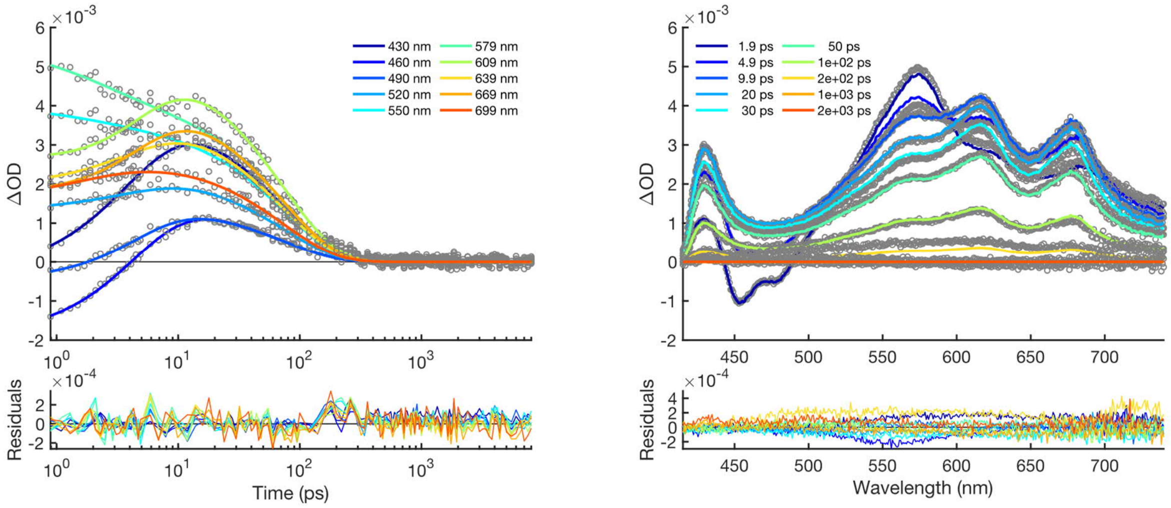

Figure S16. UV-vis TA spectroscopy of **1** in 20/80 v/v MeCN/PrCN at selected wavelengths (left) and selected times (right). Time constants obtained from global analysis are τ_1 = 1.5 ps, τ_2 = 4.1 ps, and τ_3 = 75 ps.

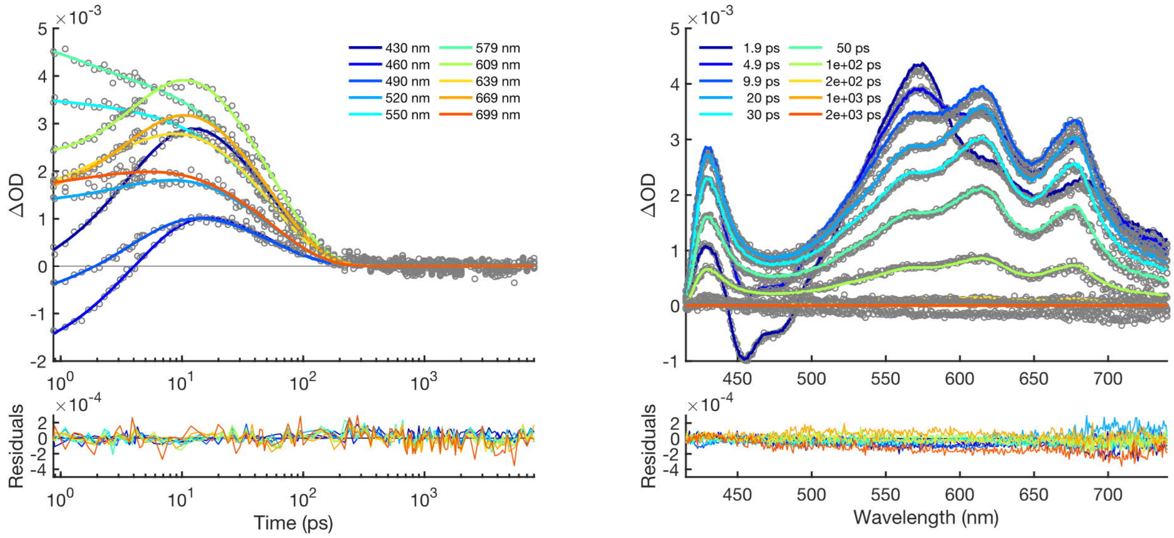

Figure S17. UV-vis TA spectroscopy of **1** in 40/60 v/v MeCN/PrCN at selected wavelengths (left) and selected times (right). Time constants obtained from global analysis are τ_1 = 1.2 ps, τ_2 = 4.4 ps, and τ_3 = 56 ps.

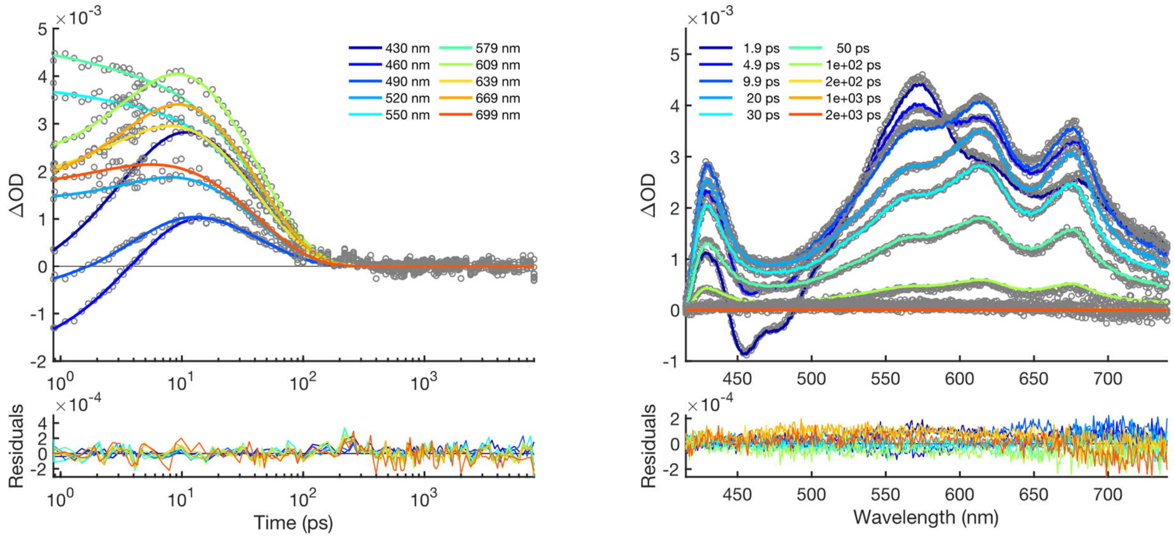

Figure S18. UV-vis TA spectroscopy of **1** in 60/40 v/v MeCN/PrCN at selected wavelengths (left) and selected times (right). Time constants obtained from global analysis are τ_1 = 1.8 ps, τ_2 = 4.2 ps, and τ_3 = 45 ps.

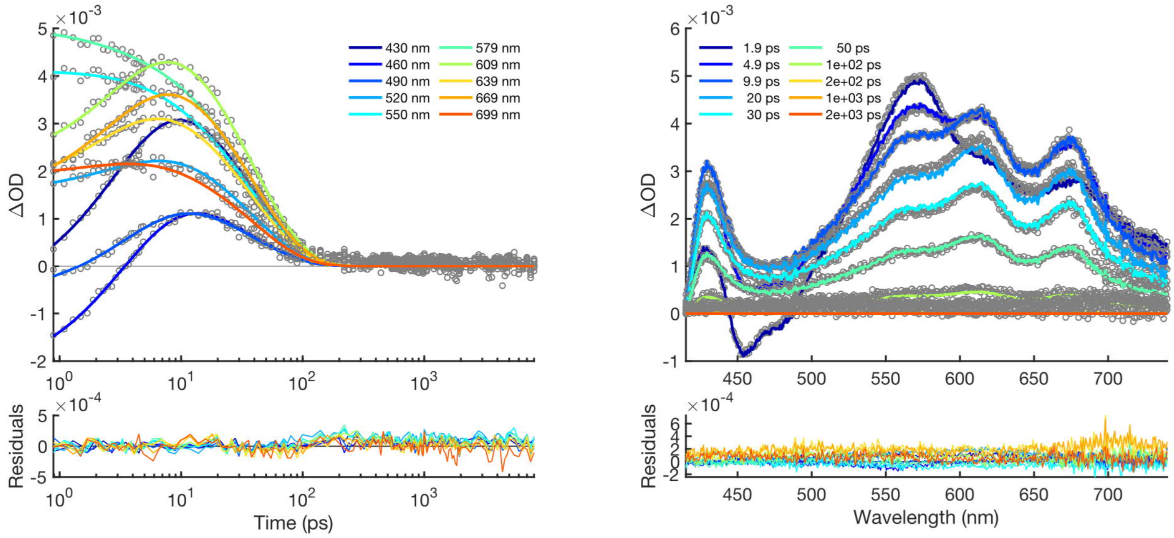

Figure S19. UV-vis TA spectroscopy of **1** in 80/20 v/v MeCN/PrCN at selected wavelengths (left) and selected times (right). Time constants obtained from global analysis are τ_1 = 2.3 ps, τ_2 = 4.1 ps, and τ_3 = 39 ps.

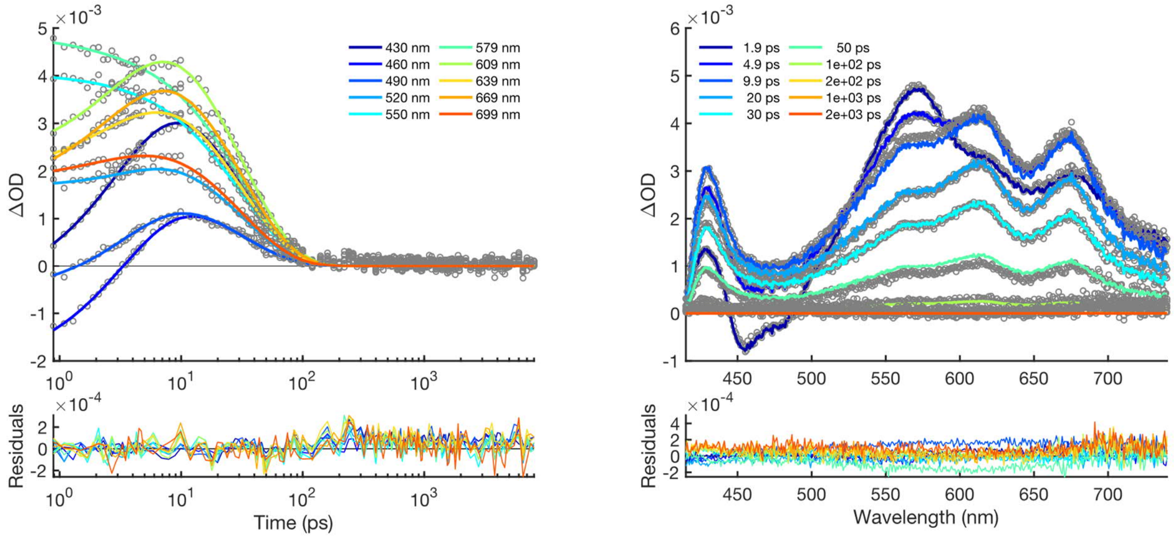

Figure S20. UV-vis TA spectroscopy of **1** in pure MeCN at selected wavelengths (left) and selected times (right). Time constants obtained from global analysis are τ_1 = 1.6 ps, τ_2 = 3.7 ps, and τ_3 = 32 ps.

1.4. Triad **2** in MeCN and PrCN Mixtures

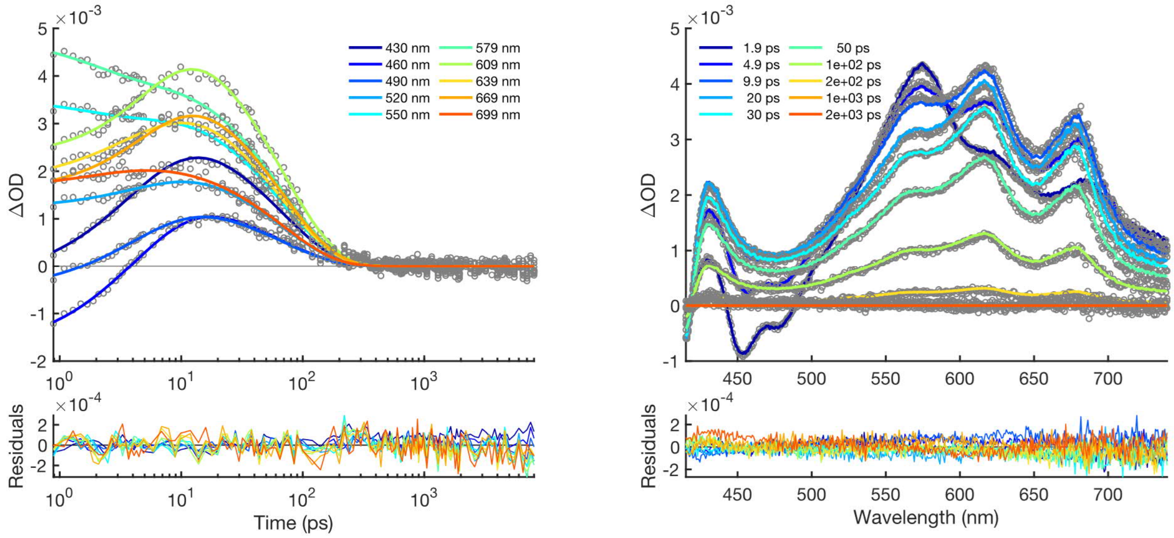

Figure S21. UV-vis TA spectroscopy of **2** in pure PrCN at selected wavelengths (left) and selected times (right). Time constants obtained from global analysis are τ_1 = 2.0 ps, τ_2 = 4.9 ps, and τ_3 = 70 ps.

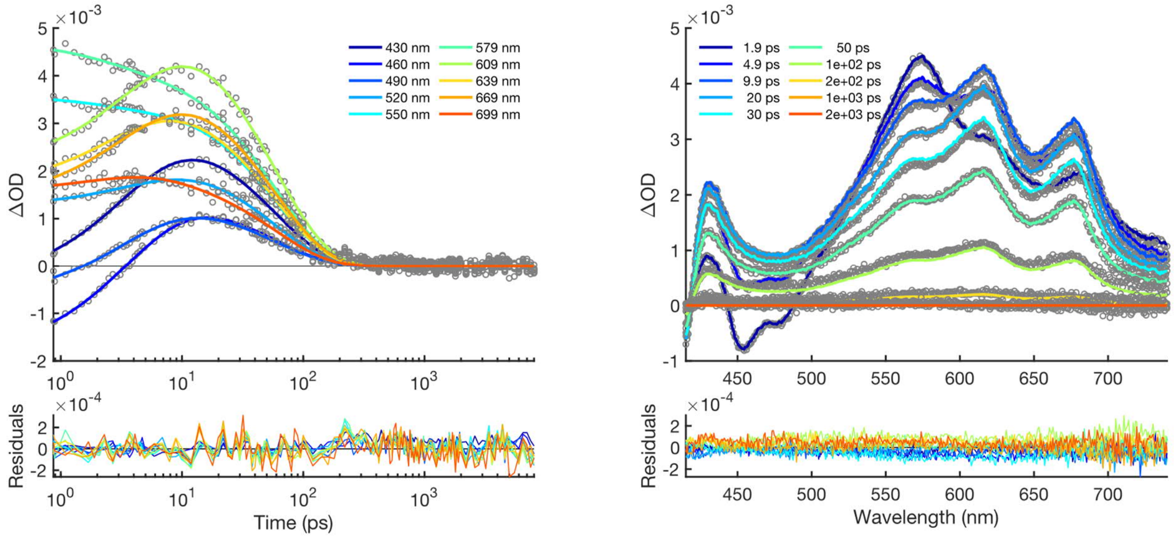

Figure S22. UV-vis TA spectroscopy of **2** in 20/80 v/v MeCN/PrCN at selected wavelengths (left) and selected times (right). Time constants obtained from global analysis are τ_1 =, 2.6 ps τ_2 = 5.4 ps, and τ_3 = 60 ps.

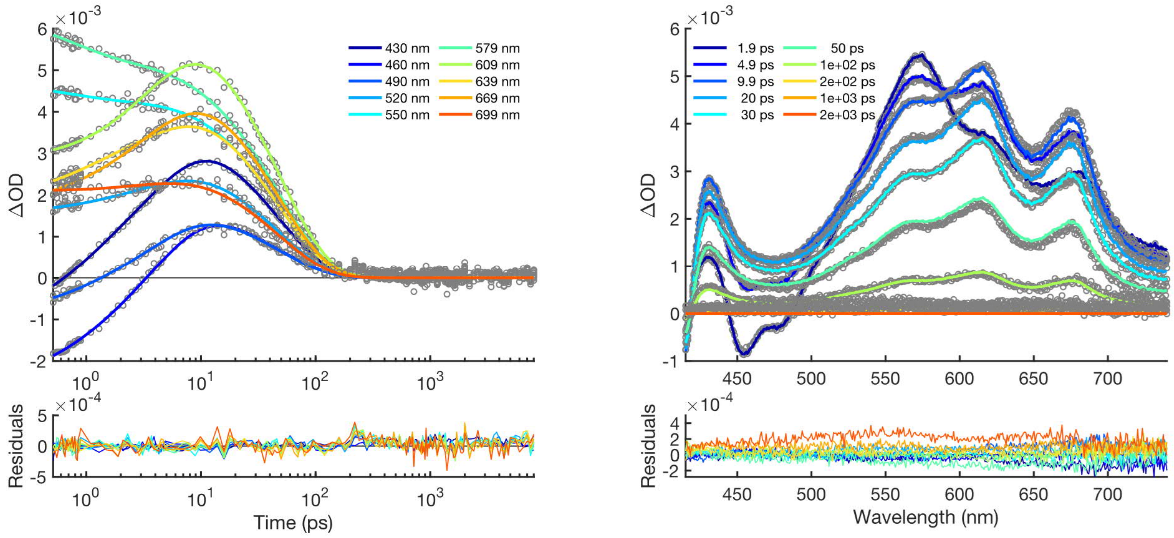

Figure S23. UV-vis TA spectroscopy of **2** in 40/60 v/v MeCN/PrCN at selected wavelengths (left) and selected times (right). Time constants obtained from global analysis are τ_1 = 0.7 ps, τ_2 = 4.1 ps, and τ_3 = 48 ps.

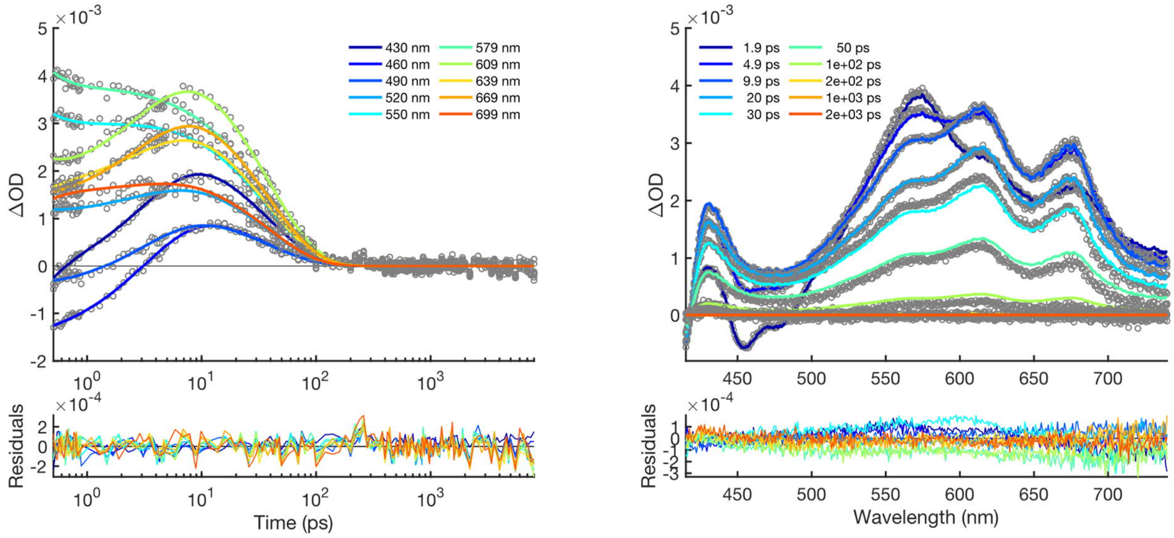

Figure S24. UV-vis TA spectroscopy of **2** in 60/40 v/v MeCN/PrCN at selected wavelengths (left) and selected times (right). Time constants obtained from global analysis are τ_1 = 0.2 ps, τ_2 = 3.4 ps, and τ_3 = 38 ps.

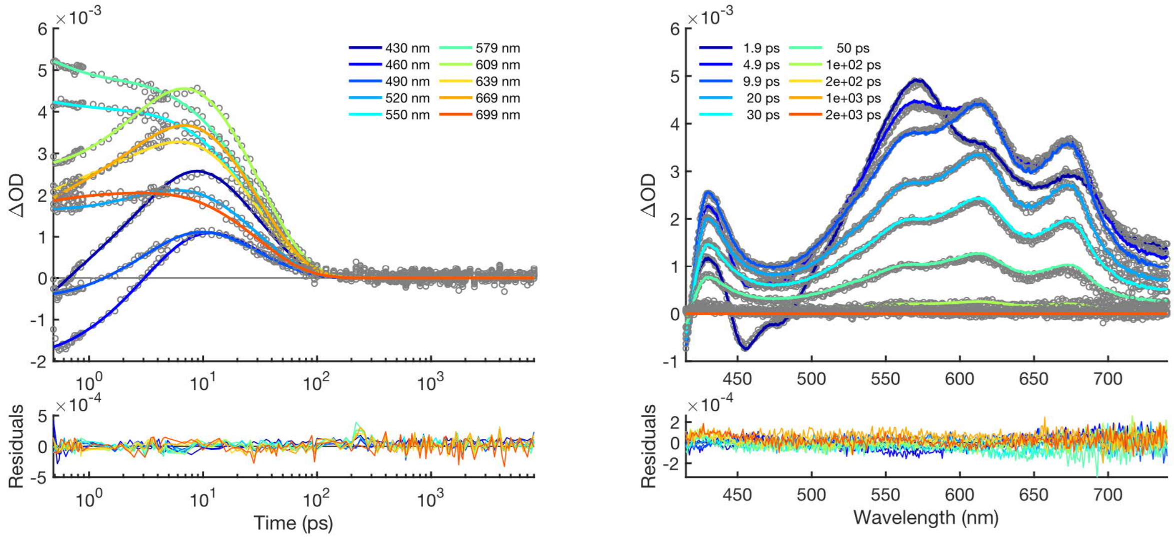

Figure S25. UV-vis TA spectroscopy of **2** in 80/20 v/v MeCN/PrCN at selected wavelengths (left) and selected times (right). Time constants obtained from global analysis are τ_1 = 0.4 ps, τ_2 = 3.4 ps, and τ_3 = 31 ps.

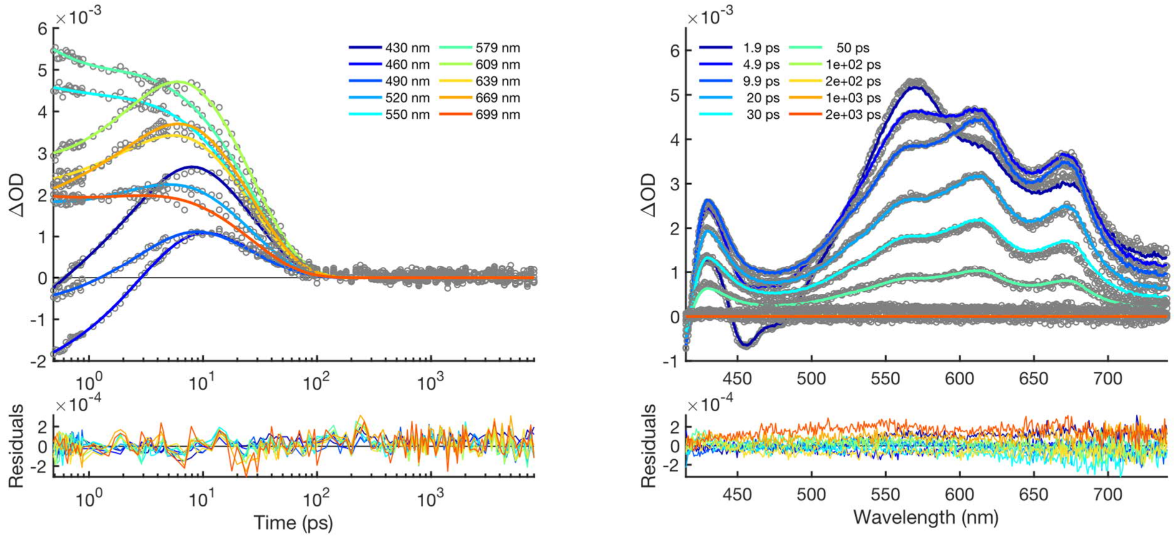

Figure S26. UV-vis TA spectroscopy of **2** in pure MeCN at selected wavelengths (left) and selected times (right). Time constants obtained from global analysis are τ_1 = 0.3 ps τ_2 = 3.1 ps, and τ_3 = 27 ps.

1.5. Triads **1** and **2** in Toluene, 298 K

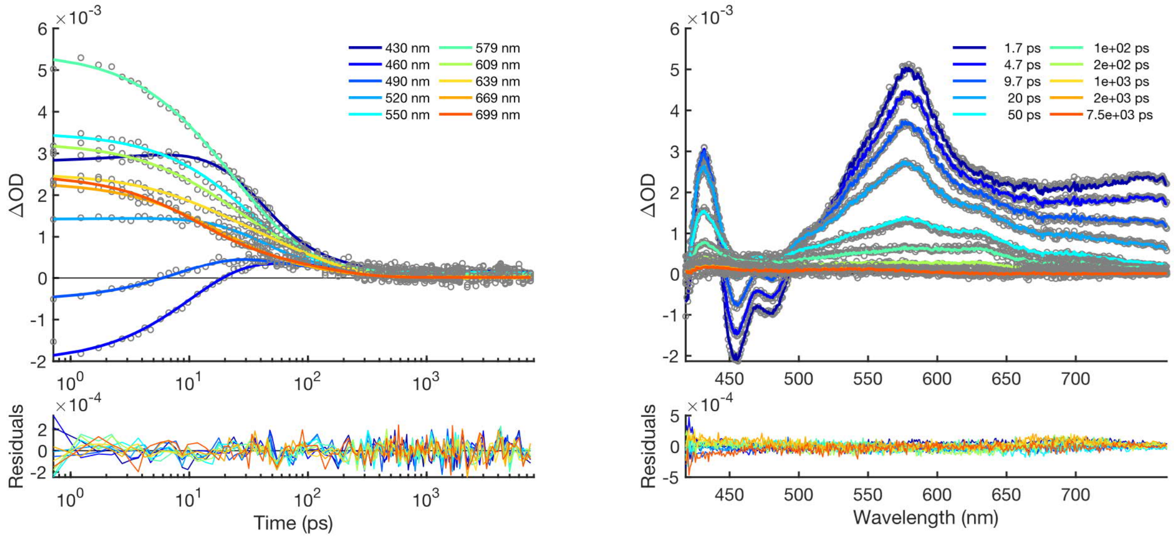

Figure S27. UV-vis TA spectroscopy of **1** in toluene at 298K at selected wavelengths (left) and selected times (right). Time constants obtained from target analysis are τ_1 = 9.7 ps, τ_2 = 32 ps, τ_3 = 140 ps, and τ_4 >> 8 ns.

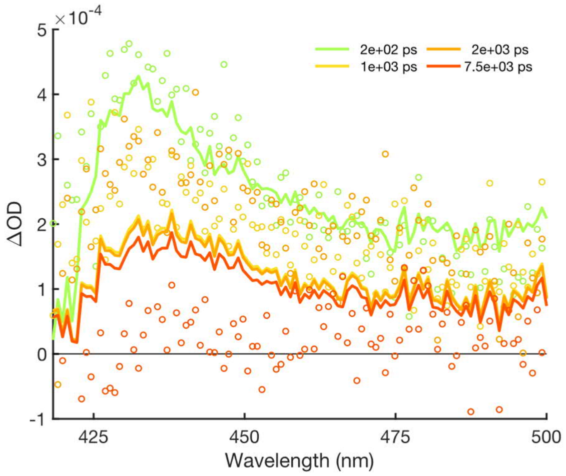

Figure S28. UV-vis TA spectroscopy of **1** in toluene at 298K at long delay times in the wavelength range where the ^{3}An peak appears. Fits are solid lines and the corresponding data points are open circles.

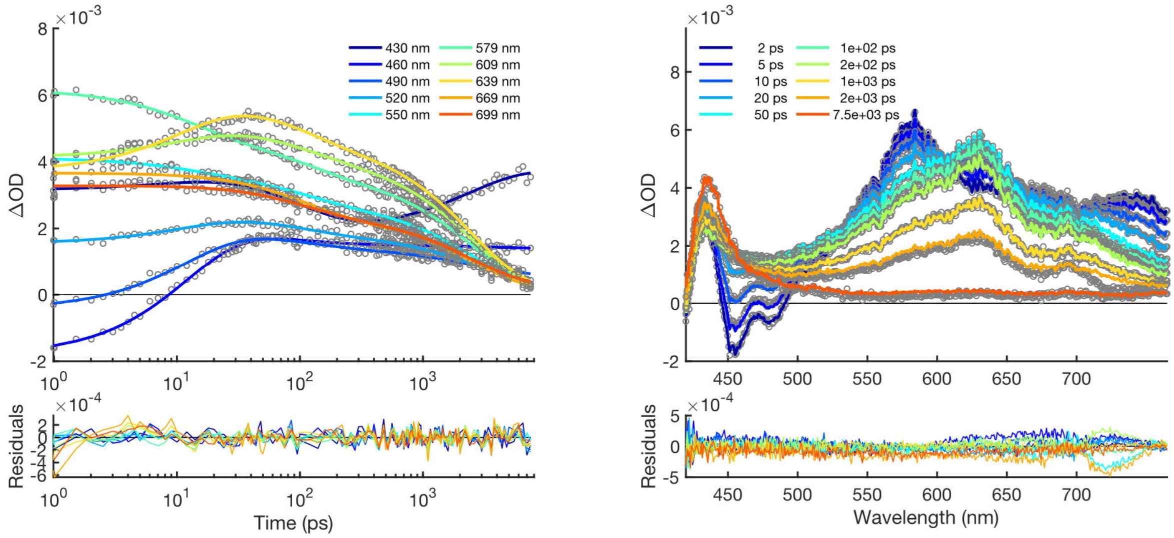

Figure S29. UV-vis TA spectroscopy of **2** in toluene at 298K at selected wavelengths (left) and selected times (right). Time constants obtained from target analysis are τ_1 = 12 ps, τ_2 = 103 ps, τ_3 = 2.5 ns, and τ_4 >> 8 ns.

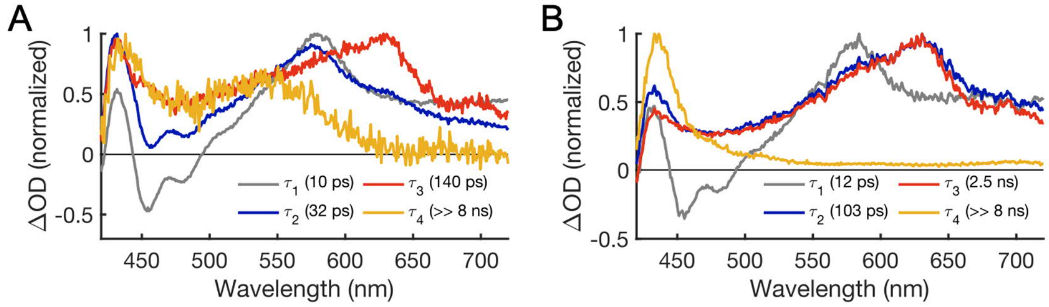

Figure S30. Normalized EAS for triads **1** (A) and **2** (B) in toluene at 298K.

1.5.1 Formation yields of the ^{1}CSS and ^{3*}An and Target Analysis.

Formation yields of the ^{1}CSS (Φ_{CSS}) and ^{3*}An (Φ_T) were estimated relative to the LES using the evolution associated spectra (EAS) amplitudes and extinction coefficients of the species involved (Table S1). The extinction coefficients were estimated from literature spectra, where $\varepsilon_{(LES,\ 580\ nm)}$ ~ 7500 cm^{-1}mol^{-1}L,[1] $\varepsilon_{(^3An,\ 425\ nm)}$ ~ 75000 cm^{-1}mol^{-1}L,[2] and $\varepsilon_{(^1CSS,\ 625\ nm)}$ ~ 7500 cm^{-1}mol^{-1}L. The latter is estimated taking $\varepsilon_{(^1GS,\ 410\ nm)}$ ~ 10000 cm^{-1}mol^{-1}L,[3,4] the concentration and absorption at 625 nm of the AN radical anion from spectro-electrochemical measurements previously reported.[5]

EAS amplitudes of LES, ^{1}CSS and ^{3}AN from global analysis were used to calculate concentrations using the aforementioned extinction coefficients. Form the concentration values, the Φ_{CSS} and Φ_T relative the LES are calculated as Φ_{CSS} = 100x[CSS]/[LES] and Φ_T = 100x[^{3}AN]/[LES]. Similarly, assuming 100% conversion yields, the apparent extinction coefficients relative to estimated values from literature result in the same Φ_{CSS} and Φ_T values (Table S1).

Table S1. Formation yields of ^{3*}An and ^{1}CSS with respect to the LES for **1** and **2** in Tol.

	^{3}An %		*^{1}CSS %*	
T (K)	**1**	**2**	**1**	**2**
298	0.5	6.8	26.0	90.3

The ^{3}AN presumably forms from the ^{3}CSS (see main text). From the spectra, 98% and 93% of the ^{1}CSS decays to the GS calculated as 100x([^{1}CSS]-[^{3}AN]) / [^{1}CSS], for **1** and **2** respectively. Formation of the ^{3}AN is a small but sizable competing path of ^{1}CSS decay. Therefore, the observed CSS decay rate corresponds to the sum of ^{3}AN formation and CR. Consistent with this interpretation, we performed a Target Analysis of the data (see Fig S31). In the fits, the parameters b1 and b2 are not floating variables. Instead, these are fix values such that 1=b1+b2 extracted from the formation yields of ^{3}An from the ^{1}CSS, such that b1=([CSS] – [^{3}An])/[CSS] and b2=1–b1. Importantly, the formation yields of the ^{1}CSS indicate a competing deactivation pathway from the LES, presumably to form the LEPT state (see main text). However, the LEPT cannot be observed in our experimental setup and therefore we are unable to determine its formation and decay kinetics. Finally, the estimate of the parameters b1 and b2 does not affect the goodness of the fit, nor the overall CSS decay rate k3 (as verified by comparison with CSS decay rates from global analysis), but it affects the CR rates (k3*b1) and ^{3}An formation rate (k3*b2). More chemically accurate models for the Target Analysis failed presumably due to data over-parametrization.

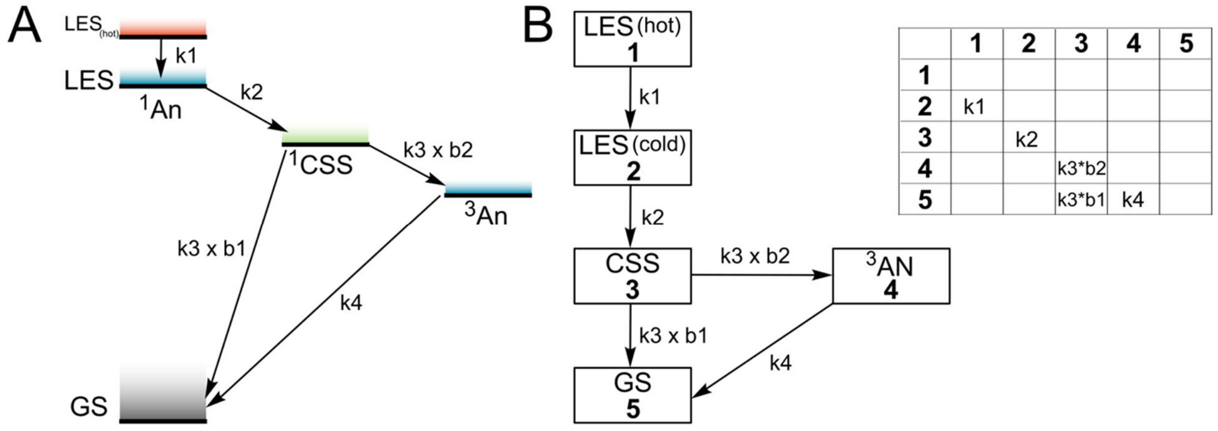

Figure S31. A) Jablonski diagram including TA observable transient species for **1** and **2** in Tol. Rate constants k1 and k2 correspond to VR and CPET-CS, respectively, k3 corresponds to CSS decay where constants b1 and b2 are weight parameters, such that: k3*b2 is the rate of triplet (^{3}An) formation and k3*b1 is the rate of MIR decay to the ground state (GS), and k4 the decay rate of ^{3}An (τ >> 8 ns, see main text). B) Compartment scheme for Target Analysis of the Tol data for **1** and **2** and corresponding K-matrix.

2. $E_T(30)$ Measurements for Butyronitrile and Acetonitrile Mixtures

The absorbance spectrum of 2,6-Diphenyl-4-(2,4,6-triphenylpyridin-1-ium-1-yl)phenolate was measured in the same solvent mixtures as used in the TA experiments. $E_T(30)$ was calculated from the absorbance maxima of the lowest energy band using equation S1, where $E_T(30)$ is in kcal/mol and λ_{max} is in nanometers. The linear relationship between mole fraction of pure solvent and $E_T(30)$ supports our use of the dielectric continuum model.

$$E_T(30) = 28591/\lambda_{max} \qquad \text{Eq. S1}$$

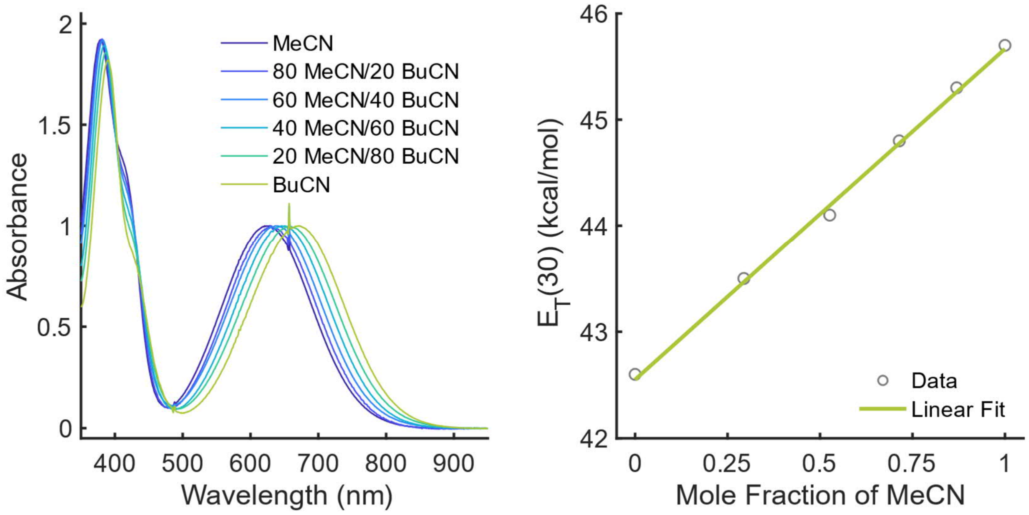

Figure S32. UV-vis absorbance spectrum (left) and calculated $E_T(30)$ parameter in MeCN and PrCN mixtures.

3. Solvent and Temperature Dependent Analysis

In the main paper it is shown that the Arrhenius analysis of the temperature data in PrCN, as well as in the nitrile mixtures, does not follow a perfect linear dependence, especially for the CR. This is suggested to arise from the inherent temperature/solvent dependencies of some of the parameters involved.

Previous work in the field has acknowledge the parameters of specific importance for the temperature dependence: the total reorganization energy (λ), the driving force ($\Delta G°$) and the activation energy (ΔG^*).[6-11] These parameters in turn depend on the static dielectric constant (ε_S) and the refractive index (n), which are equally important when regarding the studied solvent mixtures.

3.1. Marcus-Type Analysis: MeCN/PrCN Solvent Mixture

To analyze the solvent mixtures according to a Marcus-type fit (Eq. 1 and 2, main paper), the following expression was used (note T=293 K is constant):

$$\ln\left(k \cdot \sqrt{\lambda}\right) = \ln(B) - \frac{\Delta G^*}{RT} \qquad \text{Eq. S2}$$

B is the pre-exponential constant in Eq. 2 multiplied with $\sqrt{\lambda}$. To relate the rate directly to the change in the activation barrier, while going from pure MeCN to PrCN, one first have to determine the change in λ and $\Delta G°$ with the mixture.

The reorganization energy, λ, is the sum of the inner- and the outer-sphere reorganization energies, λ_{in} and λ_{out} respectively (Eq. S3). λ_{in} is here assumed to remain constant with temperature, with a computational estimate of 0.38 eV previously determined for **1** using CDFT.[5] $\lambda_{out}(T)$ on the other hand, depends on both $n(T)$ and $\varepsilon_S(T)$, given by Eq. S3:

$$\lambda = \lambda_{in} + \lambda_{out} = \lambda_{in} + \frac{e^2}{4\pi\varepsilon_0}\left(\frac{1}{2a} + \frac{1}{2b} - \frac{1}{R}\right)\left(\frac{1}{n^2} - \frac{1}{\varepsilon}\right) \qquad \text{Eq. S3}$$

where e is the elementary charge, ε_0 is the vacuum permittivity, a and b are the radii of donor and acceptor, respectively, and R is the distance between the two. Here these terms are considered independent on temperature and solvent. Its value is altered to achieve the desired λ (*vide infra*).

Thus, λ depends on the dielectric constant, ε_S, which in turn follows a linear relationship with the mole fraction of MeCN, as verified by the $E_T(30)$ experiments under section *2. $E_T(30)$ Measurements for Butyronitrile and Acetonitrile Mixtures*. Hence, going from pure MeCN (ε_S =36.64 (293K))[12] to PrCN (ε_S =24.83 (293K))[12], the ε_S is believed to alter linearly with the mole fraction. The same is assumed for both the refractive index, n (MeCN: 1.3414^{298K} recalculated to 1.3456^{293K} by Eq. S9 and ρ values from literature,[12,13] PrCN: 1.3842^{293K},[12]) and $\Delta G°$. The latter was based on the assumption that $\Delta G°(T)$, for the CS and CR respectively, is proportional to the factor $1/\varepsilon_S(T)$, calculated according Eq. S7-8, below. In the equations "ref" refers to the reference solvent, which for the solvent mixture is the PrCN solution. The input for the neat $\Delta G°_{PrCN}$ reference values for CS (**1:** -0.49 eV, **2:** -0.54 eV) were based on the CDFT calculated 0-0 transitions energy, 2.97 eV, and the computed CR driving forces for the thermalized state (**1:** -2.48 eV, **2:** -2.43 eV). For the CR the input was a weighted average of the driving forces of highest contribution according to the Tables in section 3.1.1. below.

For the fits, the $\Delta G°_{MeCN}$ values are treated as unknowns and subsequently optimized. The fixed slope in Eq.S2 of -1/RT=39.5856 (T=293K) was used as a guideline in the iterative process of finding the $\Delta G°_{MeCN}$ values. If assuming only thermalized transitions to occur ($\Delta G°_{PrCN:CS}$:-0.49

eV, $\Delta G°_{PrCN:CR}$:-2.48 eV), does not render a reasonable trend for the CR discussed in the main paper.

The goodness of fit was evaluated by examining the residual of each fit, however mostly by considering the expected trends of driving forces between MeCN and PrCN. To estimate the errors of the CR driving forces, the analyses for **1** were performed with higher (>-300 meV) or lower (<+300 meV) driving forces than the weighted average driving force of -2.06 meV, based on the contributing vibronic transitions (see main paper). Increasing the CR driving force for **1** from -2.06 meV by more than 20 meV causes the driving force in MeCN to become larger than the input value in PrCN. This was deemed intuitively faulty, although partly supported by the change in vibronic contributions going from PrCN to MeCN. Increasing the driving force further, beyond 100 meV, causes the residuals to worsen as the data points start to diverge from the straight line consequently affecting the R^2 value.

By similarly decreasing the driving force by 20 meV up to 300 meV from the weighted average, the residuals and R^2 values likewise becomes worse. Above an addition of 150 meV the deviation was considered significant. Hence, the CR driving force is believed to be -2.06±0.15 meV for triad **1**. An increase or decrease of the CR driving force by ~150 meV does not alter the conclusions drawn. The CR includes transitions to higher vibrational states as well as emphasizes the shifts in the vibronic contributions between the two solvents. Additionally, the CR barrier (115±45 meV) remains significantly higher than k_BT (~25 meV) even if the CR driving force is varied by ±150 meV in the fit.

3.1.1. Vibronic Contributions to the Charge Recombination: DCM and MeCN/PrCN mixture

In our previous study, computational efforts were made to determine the respective vibronic transitions' contributions to the overall charge recombination.[5] This calculation was performed for DCM (λ=1.4 eV), and the values are retabulated in eV in Table S2. As has already been pointed out, the 0→3 transition contributes the most to the CR in DCM. For the computations here, we assumed that the vibrational wavefunction overlaps, $S^2_{\mu v}$, as well as the relative energy gaps between the different $\Delta G°_{\mu v}$ values, are the same in the PrCN solvent. The $\Delta G°_{\mu v}$(PrCN) values however were obtained by subtracting 60 meV from the calculated values in DCM, as the computed driving forces for the CR in DCM and PrCN differed by 60 meV (see previous paper[5] and main article). Then using Eq.2 of the main text, estimations can be made of the relevant vibrational transitions for the temperature, and solvent mixtures studied here. See example in Table S3 for **1** in PrCN at 298 K, with λ=1.5 eV, and for λ=1.3 eV see main article.

Table S2. The main vibronic contributions to the CR rate constant for **1** with hydrogen at R = 2.57 Å, λ=1.4 eV in DCM (298K), going from the lowest vibrational state in the CSS (μ=0), to the vth vibrational state in the electronic GS. Adapted from [ref 5].

(μ,v)	P_μ	$\Delta G°_{\mu v}$	$\Delta G^*_{\mu v}$	$S^2_{\mu v}$	$\exp{-\Delta G^*(\mu v)}/_{RT}$	% Contrib.
0-0	1.00	-2.54	0.23	1.03E-03	1.27E-04	0.00
0-1	1.00	-2.32	0.15	4.53E-01	2.64E-03	13.12
0-2	1.00	-2.26	0.13	4.24E-01	5.63E-03	26.19
0-3	1.00	-2.12	0.09	1.03E-01	2.84E-02	31.98
0-4	1.00	-1.96	0.06	1.73E-02	1.17E-01	22.15

0-5	1.00	-1.77	0.02	1.45E-03	3.85E-01	6.11
0-6	1.00	-1.57	0.00	4.99E-05	8.26E-01	0.45
0-7	1.00	-1.34	0.00	4.78E-08	9.79E-01	0.00
0-8	1.00	-1.11	0.02	1.41E-07	5.54E-01	0.00

Table S3. The main vibronic contributions to the CR rate constant for **1** with hydrogen at R = 2.57 Å, λ=1.5 eV in PrCN (298K), going from the lowest vibrational state in the CSS (μ=0), to the vth vibrational state in the electronic GS.

(μ,v)	P_{μ}	$\Delta G^{\circ}_{\mu v}$	$\Delta G^{*}_{\mu v}$	$S^2_{\mu v}$	$\exp^{-\Delta G^{*}(\mu v)/RT}$	% Contrib.
0-0	1.00	-2.48	0.16	1.03E-03	2.06E-03	0.00
0-1	1.00	-2.26	0.10	4.53E-01	2.26E-02	21.05
0-2	1.00	-2.20	0.08	4.24E-01	4.05E-02	35.26
0-3	1.00	-2.06	0.05	1.03E-01	1.34E-01	28.47
0-4	1.00	-1.90	0.03	1.73E-02	3.62E-01	12.88
0-5	1.00	-1.71	0.01	1.45E-03	7.50E-01	2.24
0-6	1.00	-1.51	0.00	4.99E-05	1.00E+00	0.10
0-7	1.00	-1.34	0.00	4.78E-08	9.79E-01	0.00
0-8	1.00	-1.11	0.02	1.41E-07	5.54E-01	0.00

To elucidate the vibrations involved in the solvent mixture experiment, some assumptions had to be made. We assume DCM to vary similarly regarding λ and $\Delta G°$ with a fictive solvent as between MeCN and PrCN used here. Hence, λ was allowed to vary by 0.1 eV between the two solvents, and $\Delta G°_{CR}$ by 60 meV (as expected from the computations between PrCN and MeCN),[5] see Table S19 in ref. 5). Note that these calculations were all done at 298 K, and individual values later corrected to 293 K for comparison to the experimental solvent mixture study. The calculations were done for λ: 1.3 (Table S4) and 1.5 eV (Table S5).

Table S4. Vibronic transition contributions (%) with volume fraction of MeCN in the mix with PrCN, for **1**. Values for $S^2_{\mu v}$ and ΔG° were used from Table 5, together with the assumptions of λ=1.3 eV, Δλ=0.1 eV and $\Delta(\Delta G°_{CR})$=0.06 eV.

**Mol fraction MeCN**	**0-0**	**0-1**	**0-2**	**0-3**	**0-4**	**0-5**	**0-6**	**0-7**	**0-8**
**1.00**	0.00	17.73	32.02	30.56	16.22	3.29	0.17	0.00	0.00
**0.87**	0.00	16.67	30.83	31.08	17.46	3.76	0.21	0.00	0.00
**0.72**	0.00	15.39	29.31	31.59	19.02	4.42	0.27	0.00	0.00
**0.53**	0.00	13.85	27.34	32.05	21.04	5.37	0.36	0.00	0.00
**0.30**	0.00	11.96	24.73	32.29	23.69	6.82	0.51	0.00	0.00
**0.00**	0.00	9.63	21.19	31.95	27.21	9.20	0.81	0.00	0.00

Table S5. Vibronic transition contributions (%) with volume fraction of MeCN in the mix with PrCN, for **1**. Values for $S^2_{\mu v}$ and ΔG° were used from Table 5, together with the assumptions of λ=1.5 eV, Δλ=0.1 eV and $\Delta(\Delta G°_{CR})$=0.06 eV.

Mol fraction MeCN	0-0	0-1	0-2	0-3	0-4	0-5	0-6	0-7	0-8
**1.00**	0.01	29.82	41.50	21.74	6.25	0.66	0.02	0.00	0.00
**0.87**	0.01	28.77	40.96	22.60	6.87	0.77	0.02	0.00	0.00
**0.72**	0.01	27.49	40.21	23.65	7.68	0.93	0.03	0.00	0.00
**0.53**	0.01	25.88	39.17	24.94	8.80	1.16	0.04	0.00	0.00
**0.30**	0.01	23.80	37.63	26.53	10.42	1.55	0.06	0.00	0.00
**0.00**	0.00	21.05	35.26	28.47	12.88	2.24	0.10	0.00	0.00

3.2. Marcus-Type Analysis: Temperature Study

If considering the temperature dependence of λ and $\Delta G°$, and assume that it results in e.g. the overall decrease of the Marcusian barrier, the reaction will intuitively proceed faster. This would result in observed faster rates, however in the Arrhenius analysis this would not be related to any changes in the activation barrier, but instead solely correlated to the change in vibrational population distribution. To correct for this effect to the observed rates, the change in the barrier with temperature had to be estimated.

In Marcus theory the ΔG^* relates to λ and $\Delta G°$ according to Eq. S4 (Eq. 1 in the main paper).

$$\Delta G^* = \frac{(\lambda + \Delta G°)^2}{4\lambda} \qquad \text{Eq. S4}$$

The activation barrier is here simplified into a sum between a constant barrier ($\Delta G^*_{const.}$) and its temperature variation given by $\Delta(\Delta G^*)$ (see Eq. S5). With the barrier at 298 K set as the reference value, the $\Delta(\Delta G^*)$ is determined as the difference between the barrier at 298 K and the one at any other temperature (given by $\lambda(T)$ and $\Delta G°(T)$, Eq. S3 and Eq. S7-8 respectively). By rearranging the expression given by Eq. S5, the left-hand side related to the observed rates is adjusted to account for the possible changes to the activation barrier (Eq. S6). This in turn will help linearize the expression, as any change to the barrier with temperature is corrected for.

$$\ln(k) = \ln\left(\frac{C}{\sqrt{\lambda T}}\right) - \frac{(\Delta G^*_{const.} + \Delta(\Delta G^*))}{RT} \qquad \text{Eq. S5}$$

$$\ln\left(k \cdot \sqrt{\lambda T}\right) + \frac{\Delta(\Delta G^*)}{RT} = \ln(C) - \frac{\Delta G^*_{const.}}{RT} \qquad \text{Eq. S6}$$

To estimate $\Delta(\Delta G^*(T))$, the $\Delta G^*(T)$ dependence on λ and $\Delta G°$ was considered (Eq. S4). $\Delta G°(T)$ was assumed to be proportional to the factor $1/\varepsilon_S(T)$, calculated according Eq. S7-8 below. As expected for photoinduced CS and CR processes, and observed in our prior study,[5] the zwitterionic CSS is stabilized by more polar solvents (greater ε_S), thus increasing the $\Delta G°_{CS}$ (Eq. S7) and consequently decreasing $\Delta G°_{CR}$ (Eq. S9).

$$\Delta G°_{CS} = \Delta G°_{CS,ref} + \frac{e^2}{4\pi\varepsilon_0}\left(\frac{1}{2a} + \frac{1}{2b} - \frac{1}{R}\right)\left(\frac{1}{\varepsilon_s} - \frac{1}{\varepsilon_{ref}}\right) \qquad \text{Eq. S7}$$

$$\Delta G°_{CR} = \Delta G°_{CR,ref} - \frac{e^2}{4\pi\varepsilon_0}\left(\frac{1}{2a} + \frac{1}{2b} - \frac{1}{R}\right)\left(\frac{1}{\varepsilon_s} - \frac{1}{\varepsilon_{ref}}\right) \qquad \text{Eq. S8}$$

As T decreases, the ε_S and n of the solvent increases. Values for the ε_S, and n as a function of density (ρ, Eykman function, Eq. S9), for PrCN at different temperatures were taken from literature.[14-16] ρ-values for PrCN, to our knowledge, are not available for the studied T range,

therefore we assumed a linear correlation and extrapolated values from the closest available *T*-range. ρ and n are related by the empirical Eykman function:

$$\frac{n^2-1}{n+0.4} \cdot \rho\left(\frac{g}{cm^3}\right) = C_{Eykman} \quad \text{Eq. S9}$$

Knowing the n and ρ at a specific temperature makes it possible to determine the Eykman constant (C_{Eykman}), which in turn allows for the determination of various $n(T)$ values and subsequently estimations of $\lambda(T)$.

The Marcus-type analysis (Eq. S6) requires initial input-parameters, such as e.g. λ(298K) and *ΔG*°(298K), which are treated as unknowns. By making initial guesses, based on the computed values in our previous study[5] for the fully thermalized states, the *ΔG*° and λ at all other temperatures could be determined. λ(298K) was based on the computed value 1.4 eV in DCM, but several separate iterations was made were a fixed value of 1.1, 1.2, 1.3, 1.4, 1.5 and 1.6 eV was attempted. To achieve these λ values, the $\frac{e^2}{4\pi\varepsilon_0}\left(\frac{1}{2a}+\frac{1}{2b}-\frac{1}{R}\right)$ factor was varied (Eq. S3), but keeping λ_{in} constant (0.38 eV).

ΔG°(298K) was allowed to alter throughout the analysis iteratively, with the goal to ensure that $\Delta G^*_{const.}$ remained an actual constant throughout the analysis. Altering the *ΔG*° value iteratively ensured that the same $\Delta G^*_{const.}$ value was obtained from the fit (derived from the slope) as that determined from Eq. S4 at *T*=298 K. This iterative procedure allowed for the determination of *ΔG*° for both the CS and CR, presented in Table S6, for λ(298K)=1.1, 1.2, 1.3, 1.4, 1.5 and 1.6 eV. The plotted fits for all attempted λ, are shown in Fig.S33A-D. The CR plots show a clear curvature with the values seemingly plateauing above 240 K. The reason for this is discussed in the main paper.

Table S6. Fitted parameters of the Marcus analysis accounting for the temperature dependence of λ, *ΔG*° and *ΔG** in PrCN (all in eV). Below are the values when implementing an initial λ(298K) of 1.3, 1.4, 1.5 and 1.6 eV, respectively. Their corresponding *ΔG*° values for the CS and CR are given to the right.

Triad 1

°K	λ	ΔG_{CS}	ΔG_{CR}	λ	ΔG_{CS}	ΔG_{CR}	λ	ΔG_{CS}	ΔG_{CR}	λ	ΔG_{CS}	ΔG_{CR}
180	1.26	-0.50	-1.61	1.35	-0.56	-1.72	1.45	-0.62	-1.83	1.54	-0.68	-1.93
200	1.26	-0.50	-1.62	1.36	-0.55	-1.73	1.46	-0.61	-1.83	1.55	-0.67	-1.94
220	1.27	-0.49	-1.62	1.37	-0.55	-1.73	1.47	-0.60	-1.84	1.56	-0.66	-1.95
240	1.28	-0.48	-1.63	1.38	-0.54	-1.74	1.47	-0.60	-1.85	1.57	-0.65	-1.96
260	1.28	-0.48	-1.64	1.38	-0.53	-1.75	1.48	-0.59	-1.86	1.58	-0.64	-1.97
280	1.29	-0.47	-1.64	1.39	-0.53	-1.75	1.49	-0.58	-1.86	1.59	-0.64	-1.97
298	1.30	-0.47	-1.65	1.40	-0.52	-1.76	1.50	-0.58	-1.87	1.60	-0.63	-1.98

Triad 2

°K	λ	ΔG_{CS}	ΔG_{CR}	λ	ΔG_{CS}	ΔG_{CR}	λ	ΔG_{CS}	ΔG_{CR}	λ	ΔG_{CS}	ΔG_{CR}
180	1.26	-0.52	-1.61	1.35	-0.58	-1.76	1.45	-0.64	-1.87	1.54	-0.70	-1.97
200	1.26	-0.51	-1.61	1.36	-0.57	-1.76	1.46	-0.63	-1.87	1.55	-0.69	-1.98
220	1.27	-0.51	-1.62	1.37	-0.57	-1.77	1.47	-0.62	-1.88	1.56	-0.68	-1.99
240	1.28	-0.50	-1.62	1.38	-0.56	-1.77	1.47	-0.62	-1.89	1.57	-0.67	-1.99
260	1.28	-0.50	-1.62	1.38	-0.55	-1.78	1.48	-0.61	-1.89	1.58	-0.67	-2.00

280	1.29	-0.49	-1.62	1.39	-0.55	-1.79	1.49	-0.60	-1.90	1.59	-0.66	-2.01
298	1.30	-0.49	-1.62	1.40	-0.54	-1.79	1.50	-0.60	-1.91	1.60	-0.65	-2.02

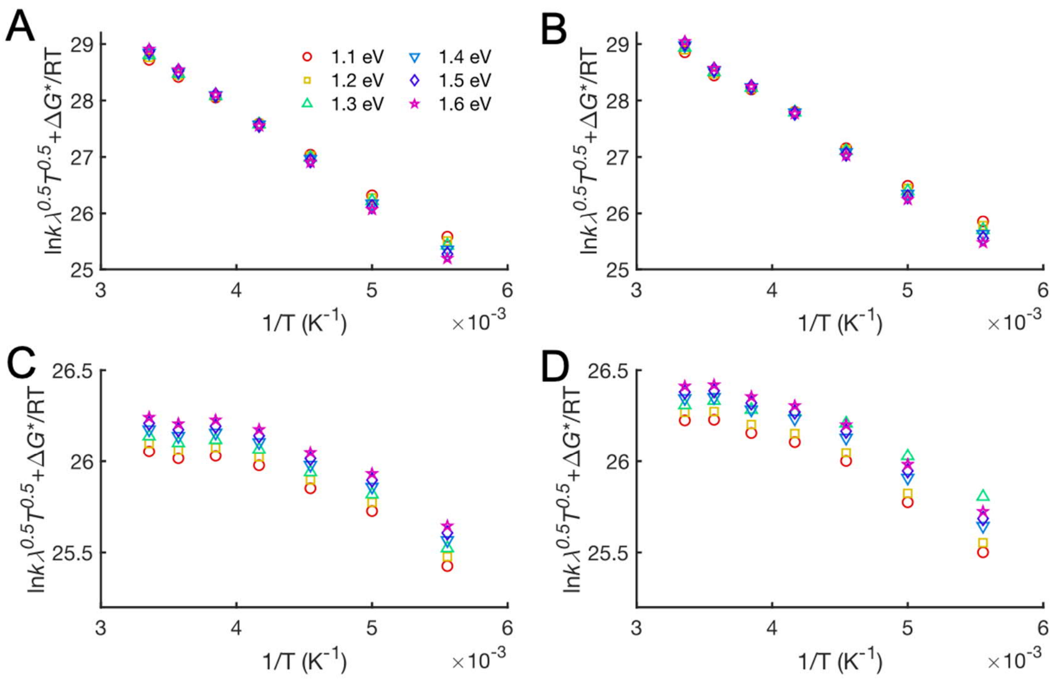

Figure S33. Marcus-type analysis in PrCN, including the temperature dependence of λ, *ΔG*° and *ΔG** and allowing λ(298K) to vary from 1.1 to 1.6 eV. Here the observed rates have been adjusted for the change in activation energy (*ΔG*(T)*). **A** and **B)** CS plots for triad **1** and **2**, respectively. **C** and **D)** CR plots for triad **1** and **2**.

3.2.1. Vibronic Contributions to the Charge Recombination: Temperature dependence

The intensity of each transition with varying temperature can simply be estimated, assuming *ΔG*° and λ, and consequently *ΔG**, to be temperature-independent. From the $S^2_{\mu\nu}$ and the exponential factor (correcting for the change in *T*), the relative vibronic contributions (%) at each temperature can be calculated.

Table S7-8 shows how the relative contributions from the different vibronic transitions (see Table 4, main paper) vary with temperature, assuming $\Delta G°_{\mu\nu}$ and λ (as well as $S_{\mu\nu}$) are constant; constant $\Delta G°_{\mu\nu}$ and λ corresponds to the fits according to Eq. S6, where the term $\Delta(\Delta G^*)$ is used to correct for their temperature dependence. Indeed, at higher temperatures, the transitions with larger $\Delta G^*_{\mu\nu}$ and larger vibrational overlap ($S_{\mu\nu}$) become more important. At 180 K, the main contributions are from the 0→4 transition, while at 298 K the main contribution is from the 0→3 transition.

Table S7. Relative contributions (%) to k_{CR} of the most important vibronic transitions at different temperatures, for **1** in PrCN, as calculated from computational data in Table 4 and λ=1.3 eV.

T(K)/ (u,v)	0-0	0-1	0-2	0-3	0-4	0-5	0-6	0-7	0-8
**298**	0.00	9.62	21.17	31.95	27.23	9.21	0.82	0.00	0.00

**280**	0.00	7.99	18.57	31.53	29.83	11.04	1.04	0.00	0.00
**260**	0.00	6.19	15.44	30.44	32.89	13.64	1.39	0.00	0.00
**240**	0.00	4.49	12.14	28.49	35.95	17.04	1.90	0.00	0.00
**220**	0.00	2.96	8.83	25.48	38.63	21.43	2.65	0.00	0.01
**200**	0.00	1.73	5.78	21.34	40.32	27.02	3.80	0.01	0.01
**180**	0.00	0.84	3.25	16.25	40.16	33.91	5.58	0.01	0.01

Table S8. Relative contribution (%) to k_{CR} of the most important vibronic transitions at different temperatures, for **1** in PrCN, as calculated from computational data in Table 4 though with λ=1.5 eV.

T(K)/(μ,ν)	0-0	0-1	0-2	0-3	0-4	0-5	0-6	0-7	0-8
**298**	0.00	21.04	35.25	28.47	12.89	2.24	0.10	0.00	0.00
**280**	0.00	19.50	33.91	29.59	14.27	2.60	0.12	0.00	0.00
**260**	0.00	17.59	32.09	30.89	16.16	3.12	0.15	0.00	0.00
**240**	0.00	15.46	29.81	32.19	18.51	3.83	0.19	0.00	0.00
**220**	0.00	13.10	26.99	33.37	21.46	4.83	0.25	0.00	0.00
**200**	0.00	10.55	23.51	34.21	25.16	6.24	0.33	0.00	0.00
**180**	0.00	7.87	19.32	34.30	29.73	8.32	0.46	0.00	0.00

3.2.2. Matyushov Model – Linearizing the temperature dependence.

The continuum model used in the Marcus-type analysis, attributes all solvent effects onto two parameters: *n* and *ε*, of which we have accounted for to the best of our knowledge. Nonetheless it still gives unsatisfactory results. In an attempt to improve the analysis, the *Molecular Model* published by Matyushov and coworkers in 1999,[17] was implemented. They had fitted an intramolecular CR in MeCN, which is similar to our system. One especially noticeable observation is the change in sign of the slope for the solvent reorganization with temperature. We attempt to utilize their observed temperature dependencies ($d\lambda/d$T and $d\Delta$G°/dT) for **1** in PrCN, within the previously described Marcus type analysis procedure. We note that the slopes provided are for another system as well as in another solvent (MeCN) than the PrCN used for our measurements. Additionally, we assume the same temperature dependence for the CS reaction, however the opposite sign for the driving force. The results can be found in Table S9-10, where we have chosen to apply different percentages of the *Molecular Model* slopes. If indicated with 100% for both λ_{out} and ΔG°, it means the analysis was performed with the full slopes given by Matyushov *et al.*. A lower percentage indicates that we applied a shallower temperature dependence. Most of the fits were performed with a fixed input total reorganization energy at 298 K (with λ_{in}=0.38 eV), which then was allowed to vary with temperature, by the obtained slope for λ_{out}. In two cases however, λ was instead fitted, with the driving forces (CS and CR) fixed to the values computed in our previous publication; -0.49 and -2.48 eV for **1** and -0.54 and -2.43 for **2**.[5]

The *Molecular Model* linearizes the fits in all cases, however not all attempts results in reasonable values. The full *Molecular Model* clearly give the most linear fits, however the pre-exponential factor, *C*, is larger than the theoretical limit of ~$1*10^{13}$ s^{-1}. Going to lower percentages of the slopes,

the value of *C* becomes more reasonable, though still remaining high for the CS. Additionally, in some cases the collective driving forces vastly exceeded that of the E_{00} (2.97 eV). The fits subjected to the least questionable results have been marked with an outlined box.

Table S9. The results at 298 K of the applied linear temperature dependence given by the Matyushov and coworkers' *Molecular Model*, for the solvent reorganization energy and CR the driving force in **1**. The slopes obtain from Matyushovs' model were either kept identical or lowered to predict a smaller temperature dependence (50, 60, 75%). A combination of different slope percentages were also examined. Note that the bold values refers to the fixed values at 298 K, in the analysis and italics refers to questionable (of various degrees) values. Two attempts were made where the reorganization energy was fitted while the driving forces (CS and CR) were locked. The results in the black boxes are considered the best results.

%Slope$_{\Delta G°}$	%Slope$_{\lambda out}$	$\Delta G°_{CS}$	$\Delta G°_{CR}$	$\lambda_{CS/CR}$	C_{CS}	C_{CR}	$\Delta G^*_{CS/CR}$	$R^2_{CS/CR}$
100	100	*-0.31*	-2.44	**1.2**	*1.1E+14*	*2.7E+15*	0.17/0.32	0.9998/0.9999
100	100	*-0.38*	-2.54	**1.3**	*8.8E+13*	*1.0E+15*	0.16/0.29	0.9997/0.9999
100	100	*-0.46*	*-2.64*	**1.4**	*7.4E+13*	*4.7E+14*	0.16/0.27	0.9997/0.9999
100	100	*-0.54*	*-2.74*	**1.5**	*6.3E+13*	*2.4E+14*	0.15/0.26	0.9997/0.9999
100	100	*-0.62*	*-2.84*	**1.6**	*5.5E+13*	*1.4E+14*	0.15/0.24	0.9997/0.9998
75	75	*-0.38*	-2.17	**1.2**	*3.9E+13*	*2.3E+13*	0.14/0.20	0.9996/0.9996
75	75	-0.46	-2.27	**1.3**	*3.4E+13*	1.4E+13	0.14/0.18	0.9996/0.9994
75	75	-0.53	-2.38	**1.4**	*3.1E+13*	9.3E+12	0.13/0.17	0.9996/0.9992
75	75	*-0.61*	-2.49	**1.5**	2.8E+13	6.6E+12	0.13/0.16	0.9995/0.9991
60	60	-0.42	-2.02	**1.2**	2.3E+13	2.8E+12	0.13/0.14	0.9995/0.9984
60	60	-0.50	-2.13	**1.3**	2.1E+13	2.1E+12	0.12/0.13	0.9991/0.9981
60	60	*-0.57*	-2.24	**1.4**	1.9E+13	1.6E+12	0.12/0.13	0.9994/0.9977
60	60	*-0.65*	-2.35	**1.5**	1.8E+13	1.3E+12	0.12/0.12	0.9994/0.9974
60	60	*-0.73*	*-2.46*	**1.6**	1.7E+13	1.0E+12	0.12/0.11	0.9994/0.9970
50	50	-0.45	-1.93	**1.2**	1.7E+13	9.0E+11	0.12/0.11	0.9994/0.9967
50	50	-0.52	-2.04	**1.3**	1.5E+13	7.2E+11	0.12/0.11	0.9994/0.9961
50	50	*-0.60*	-2.15	**1.4**	1.4E+13	6.0E+11	0.11/0.10	0.9993/0.9956
50	50	*-0.68*	-2.26	**1.5**	1.4E+13	5.2E+11	0.11/0.10	0.9993/0.9951
50	75	*-0.64*	-2.24	**1.4**	9.3E+12	1.5E+12	0.10/0.13	0.9991/0.9977
75	50	-0.49	-2.29	**1.4**	*5.1E+13*	2.8E+12	0.15/0.14	0.9997/0.9984
60	60	**-0.49**	**-2.48**	*1.29/1.62*	2.1E+13	1.0E+12	0.12/0.11	0.9995/0.9969
100	100	**-0.49**	**-2.48**	*1.43/1.24*	*7.0E+13*	*1.7E+15*	0.16/0.31	0.9997/0.9999

Table S10. The results at 298 K of the applied linear temperature dependence given by the Matyushov and coworkers' *Molecular Model*, for the solvent reorganization energy and CR the driving force in **2**. The slopes obtain from Matyushovs' model were either kept identical or lowered to predict a smaller temperature dependence (50, 60, 75%). A combination of different slope percentages were also examined. Note that the bold values refers to the "fixed" values at 298 K, in the analysis and italics refers to questionable (of various degrees) values. Two attempts were

made where the reorganization energy was fitted while the driving forces (CS and CR) were locked. The results in the black boxes are considered the best results.

%Slope$_{\Delta G°}$	%Slope$_{\lambda out}$	$\Delta G°_{CS}$	$\Delta G°_{CR}$	$\lambda_{CS/CR}$	C_{CS}	C_{CR}	$\Delta G^*_{CS/CR}$	R^2$_{CS/CR}$
100	100	*-0.33*	-2.46	**1.2**	*8.8E+13*	*5.3E+15*	0.16/0.33	0.9979/0.9999
100	100	*-0.41*	-2.56	**1.3**	*7.2E+13*	*2.0E+15*	0.15/0.31	0.9978/0.9999
100	100	*-0.49*	*-2.67*	**1.4**	*6.0E+13*	*9.2E+14*	0.15/0.29	0.9977/0.9999
100	100	*-0.57*	*-2.77*	**1.5**	*5.2E+13*	*4.7E+14*	0.14/0.27	0.9975/0.9999
100	100	*-0.65*	*-2.87*	**1.6**	*4.6E+13*	*2.6E+14*	0.14/0.25	0.9974/0.9999
75	75	*-0.40*	-2.20	**1.2**	*3.3E+13*	*4.4E+13*	0.13/0.21	0.9971/0.9997
75	75	-0.48	-2.30	**1.3**	2.9E+13	2.6E+13	0.13/0.19	0.9969/0.9996
75	75	-0.56	-2.41	**1.4**	2.6E+13	1.7E+13	0.13/0.18	0.9968/0.9995
75	75	*-0.64*	*-2.52*	**1.5**	2.3E+13	1.2E+13	0.12/0.17	0.9967/0.9993
60	60	*-0.44*	-2.05	**1.2**	1.9E+13	5.1E+12	0.12/0.15	0.9964/0.9989
60	60	-0.52	-2.16	**1.3**	1.8E+13	3.7E+12	0.12/0.14	0.9963/0.9986
60	60	-0.57	-2.27	**1.4**	1.7E+13	2.9E+12	0.12/0.14	0.9960/0.9981
60	60	*-0.67*	*-2.38*	**1.5**	1.5E+13	2.3E+12	0.11/0.13	0.9962/0.9983
60	60	*-0.75*	*-2.49*	**1.6**	1.4E+13	1.9E+12	0.11/0.12	0.9959/0.9978
50	50	-0.47	-1.96	**1.2**	1.4E+13	1.6E+12	0.11/0.12	0.9958/0.9976
50	50	-0.54	-2.07	**1.3**	1.3E+13	1.3E+12	0.11/0.11	0.9957/0.9972
50	50	*-0.62*	-2.19	**1.4**	1.2E+13	1.1E+12	0.11/0.11	0.9956/0.9968
50	50	*-0.70*	-2.30	**1.5**	1.2E+13	9.0E+11	0.11/0.11	0.9955/0.9964
50	75	*-0.66*	-2.27	**1.4**	8.1E+12	2.8E+12	0.097/0.13	0.9945/0.9984
75	50	-0.51	-2.32	**1.4**	*4.3E+13*	5.1E+12	0.14/0.15	0.9974/0.9988
60	60	**-0.54**	**-2.43**	*1.33/1.54*	1.7E+13	2.1E+12	0.12/0.13	0.9962/0.9980
100	100	**-0.54**	**-2.43**	*1.47/1.17*	*5.4E+13*	*7.5E+15*	0.15/0.34	0.9976/0.9999

1. Bachilo, S. M. Absorption from excited singlet states of anthracene and 1,2-benzanthracene. *J. Appl. Spectrosc.* **1993,** *58*, 99-103.
2. Dempster, D. N.; Morrow, T.; Quinn, M. F. Extinction coefficients for triplet-triplet absorption in ethanol solutions of anthracene, naphthalene, 2,5-diphenyloxazole, 7-diethylamino-4-methyl coumarin and 4-methyl-7-amino-carbostyril. *J. Photochem.* **1973,** *2*, 329-341.
3. Jones, R. N. The Ultraviolet Absorption Spectra of Anthracene Derivatives. *Chem. Rev.* **1947,** *41*, 353-371.
4. Glöcklhofer, F.; Rosspeintner, A.; Pasitsuparoad, P.; Eder, S.; Fröhlich, J.; Angulo, G.; Vauthey, E.; Plasser, F. Effect of symmetric and asymmetric substitution on the optoelectronic properties of 9,10-dicyanoanthracene. *Mol. Syst. Des. Eng.* **2019,** *4*, 951-961.
5. Parada, G. A.; Goldsmith, Z. K.; Kolmar, S.; Pettersson Rimgard, B.; Mercado, B. Q.; Hammarström, L.; Hammes-Schiffer, S.; Mayer, J. M. Concerted proton-electron transfer reactions in the Marcus inverted region. *Science* **2019,** *364*, 471.

6. Closs, G. L.; Johnson, M. D.; Miller, J. R.; Piotrowiak, P. A connection between intramolecular long-range electron, hole, and triplet energy transfers. *J. Am. Chem. Soc.* **1989,** *111*, 3751-3753.
7. Liang, N.; Miller, J. R.; Closs, G. L. Temperature-independent long-range electron transfer reactions in the Marcus inverted region. *J. Am. Chem. Soc.* **1990,** *112*, 5353-5354.
8. Finckh, P.; Heitele, H.; Volk, M.; Michel-Beyerle, M. E. Electron donor/acceptor interaction and reorganization parameters from temperature-dependent intramolecular electron-transfer rates. *J. Phys. Chem.* **1988,** *92*, 6584-6590.
9. Liu, J. Y.; Bolton, J. R. Intramolecular photochemical electron transfer. 7. Temperature dependence of electron-transfer rates in covalently linked porphyrin-amide-quinone molecules. *J. Phys. Chem.* **1992,** *96*, 1718-1725.
10. Kroon, J.; Oevering, H.; Verhoeven, J. W.; Warman, J. M.; Oliver, A. M.; Paddon-Row, M. N. Temperature effects on intramolecular electron transfer kinetics under "normal", "inverted", and "nearly optimal" conditions. *J. Phys. Chem.* **1993,** *97*, 5065-5069.
11. Serpa, C.; Gomes, P. J. S.; Arnaut, L. G.; de Melo, J. S.; Formosinho, S. J. Temperature Dependence of Ultra-Exothermic Charge Recombinations. *ChemPhysChem* **2006,** *7*, 2533-2539.
12. *CRC Handbook of Chemistry and Physics, Internet Version 2005*. Lide, D. R., Ed. CRC Press: Boca Raton, FL, 2005.
13. Martins, F.; Leitão, R. E.; Nunes, N. Volumetric and refractive index study of the ternary mixture methanol/formamide/acetonitrile at 298.15K. *J. Mol. Liq.* **2017,** *234*, 463-468.
14. Michnick, R. B.; Rhoads, K. G.; Sadoway, D. R. Relative Dielectric Constant Measurements in the Butyronitrile-Chloroethane System at Subambient Temperatures. *J. Electrochem. Soc.* **1997,** *144*, 2392-2398.
15. Jannelli, L.; Lopez, A.; Silvestri, L. Thermodynamic and physical properties of binary mixtures involving sulfolane. 2. Excess dielectric constants of mixing sulfolane and propionitrile, butyronitrile and valeronitrile. *J. Chem. Eng. Data* **1983,** *28*, 166-169.
16. Grande, M. d. C.; Álvarez Juliá, J.; Barrero, C. R.; Marschoff, C. M. Density, speed of sound, refractive index and related properties of the binary mixture ethyl lactate + butyronitrile at various temperatures. *Phys. Chem. Liq.* **2020**, 1-13.
17. Vath, P.; Zimmt, M. B.; Matyushov, D. V.; Voth, G. A. A Failure of Continuum Theory: Temperature Dependence of the Solvent Reorganization Energy of Electron Transfer in Highly Polar Solvents. *J. Phys. Chem. B* **1999,** *103*, 9130-9140.
